# Natural variation in *ZmNRT2.5* modulates husk leaf width and promotes seed protein content in maize

**DOI:** 10.1111/pbi.14559

**Published:** 2025-01-05

**Authors:** Qi Wang, Min Wang, Ai‐Ai Xia, Jin‐Yu Wang, Zi Wang, Tao Xu, De‐Tao Jia, Ming Lu, Wei‐Ming Tan, Jin‐Hong Luo, Yan He

**Affiliations:** ^1^ College of Agronomy and Biotechnology China Agricultural University China; ^2^ National Key Laboratory of Plant Molecular Genetics (NKLPMG), CAS Center for Excellence in Molecular Plant Sciences (CEMPS) Chinese Academy of Sciences Shanghai China; ^3^ Key Laboratory of Seed Innovation, Institute of Genetics and Developmental Biology Chinese Academy of Sciences Beijing China; ^4^ Tieling Academy of Agricultural Sciences Tieling China; ^5^ Maize Research Institute Jilin Academy of Agricultural Sciences Gongzhuling China

**Keywords:** maize, GWAS, husk leaf width, seed protein content, *ZmNRT2.5*

## Abstract

The husk leaf of maize (*Zea mays*) encases the ear as a modified leaf and plays pivotal roles in protecting the ear from pathogen infection, translocating nutrition for grains and warranting grain yield. However, the natural genetic basis for variation in husk leaf width remains largely unexplored. Here, we performed a genome‐wide association study for maize husk leaf width and identified a 3‐bp InDel (insertion/deletion) in the coding region of the nitrate transporter gene *ZmNRT2.5*. This polymorphism altered the interaction strength of ZmNRT2.5 with another transporter, ZmNPF5, thereby contributing to variation in husk leaf width. We also isolated loss‐of‐function mutants in *ZmNRT2.5*, which exhibited a substantial decrease in husk leaf width relative to their controls. We demonstrate that ZmNRT2.5 facilitates the transport of nitrate from husk leaves to maize kernels in plants grown under low‐nitrogen conditions, contributing to the accumulation of proteins in maize seeds. Together, our findings uncovered a key gene controlling maize husk leaf width and nitrate transport from husk leaves to kernels. Identification of the *ZmNRT2.5* loci offers direct targets for improving the protein content of maize seeds via molecular‐assisted maize breeding.

## Introduction

Maize (*Zea mays*) husk leaf, as a part of maize stover, attaches to the ear shank and encases the ear as a modified leaf (Nwanya *et al*., 2019). Therefore, husks play important biological roles by protecting the ear from pathogen infection or pest damage and contributing to maize yield (Betrán and Isakeit, 2004; Cao *et al*., 2014; Demissie *et al*., 2008; Widstrom *et al*., 2003). Indeed, similar to regular leaves, the husk leaf can produce carbohydrates through photosynthesis that provide energy for kernel development (Pengelly *et al*., 2011; Wang *et al*., 2013). Furthermore, the husk leaf exhibits a higher conversion efficiency for photosynthetic products than other leaves with the same surface area, which contributes to ear development (Cantrell and Geadelmann, 1981; Fujita *et al*., 1995). The husk leaf also serves as a temporary storage for nutrients from other tissues (Ogunbosoye and Odedire, 2022; Osagie *et al*., 2017; Riaz *et al*., 2021). While husk leaves are crucial for maize ear development, most husk leaf studies have focused on phenotypic and physiological aspects, with limited work exploring the genetic basis of husk leaf development.

Husk leaf development originates from the lateral meristem, and the primary traits that shape husk leaf function are husk length, husk width, husk thickness and husk layer number (Wang *et al*., 2013). Genome‐wide association studies (GWAS) have been performed for husk leaf traits using large‐scale populations with high marker density, leading to the identification of several genomic regions associated with each trait (Cui *et al*., 2018; Jiang *et al*., 2020; Zhang *et al*., 2021). For example, a GWAS by Zhou *et al*. (2016) identified eight single‐nucleotide polymorphisms (SNPs) for husk leaf number, while an independent GWAS by Zhou *et al*. (2016) detected nine significant SNPs for husk leaf weight. In addition, a GWAS performed by Cui *et al*. (2016) revealed nine polymorphisms significantly associated with four husk leaf traits. A major‐effect quantitative trait locus (QTL) for husk leaf number, qHN7, was fine‐mapped to a genomic interval containing four genes associated with plant growth and development (Zhou *et al*., 2020). *Zea mays Methyltransferase 2* (*ZMET2*), encoding a DNA methyltransferase and negatively modulating the number of husk layers in maize, was identified during an association study that explored the potential contribution of seven DNA methyltransferase genes and four DNA demethylase genes to variation in 19 agronomic traits, using a maize association panel consisting of 508 inbred lines (Wang *et al*., 2024). Husk leaf width is an important trait that plays an important role in ear development (Crane *et al*., 1959; Shufang *et al*., 2014). We previously identified the gene *Regulator of Husk leaf Width 1* (*RHW1*) by GWAS, which positively regulates husk leaf width at a very early stage of husk leaf development in maize (Xia *et al*., 2023).

Nitrogen is an essential nutrient required for plant growth and development. Nitrate (NO_3_^−^) serves as an important nitrogen source and functions as a signalling molecule, initiating various aspects of plant physiology, growth and development throughout the plant life cycle (Liu *et al*., 2022). Once taken up by the roots, nitrate can be transported to the leaves and seeds (Chopin *et al*., 2007a, 2007b). There are seven *NITRATE TRANSPORTER 2* (*NRT2*) genes in Arabidopsis (*Arabidopsis thaliana*), and their encoded proteins function in the high‐affinity transport system (HATS) (Krapp *et al*., 2014; Orsel and Anne Krapp, 2002). In Arabidopsis, four NRT2 transporters (AtNRT2.1, AtNRT2.2, AtNRT2.3 and AtNRT2.4) are involved in nitrate uptake (Deng *et al*., 2023; Lezhneva *et al*., 2014). AtNRT2.5, another member of the NRT2 family, was also reported to be involved in nitrate acquisition and remobilisation in Arabidopsis under nitrogen‐limited conditions (Lezhneva *et al*., 2014). The maize genome has seven *NRT2* genes, whose expression levels vary across different tissues and developmental stages, with some *ZmNRT*s being exclusively expressed in particular tissues or developmental stages. Furthermore, the expression profile of *ZmNRT2.5* was shown to vary in response to nitrogen level among diverse maize inbred lines with different nitrogen uptake rates (Jia *et al*., 2023). *ZmNRT2.5* expression responds quickly to nitrogen starvation and is expressed at low levels across all organs at the V7 stage, with exclusively high expression levels in the husk leaf (Dechorgnat *et al*., 2019).

Maize husk leaves are crucial for the distribution of nitrogen during the grain‐filling period (Fujita *et al*., 1995). In this study, we performed GWAS for maize third husk leaf width using a diverse maize association panel and demonstrated that a 3‐bp InDel (InDel1604) in the end of the *ZmNRT2.5* coding region is a major causative polymorphism for natural variation in husk leaf width that functions by affecting the interaction strength between ZmNRT2.5 and another nitrate transporter, ZmNPF5. We revealed that *ZmNRT2.5* encodes a nitrate transporter, facilitating the transport of nitrate from husk leaves to maize kernels and thus accumulating the protein content of maize seeds. Taken together, our results demonstrate that *ZmNRT2.5* provides an effective tool for improving the protein content of maize seeds by marker‐assisted breeding.

## Results

### *ZmNRT2.5* is associated with maize husk leaf width variation

**Figure 1:**
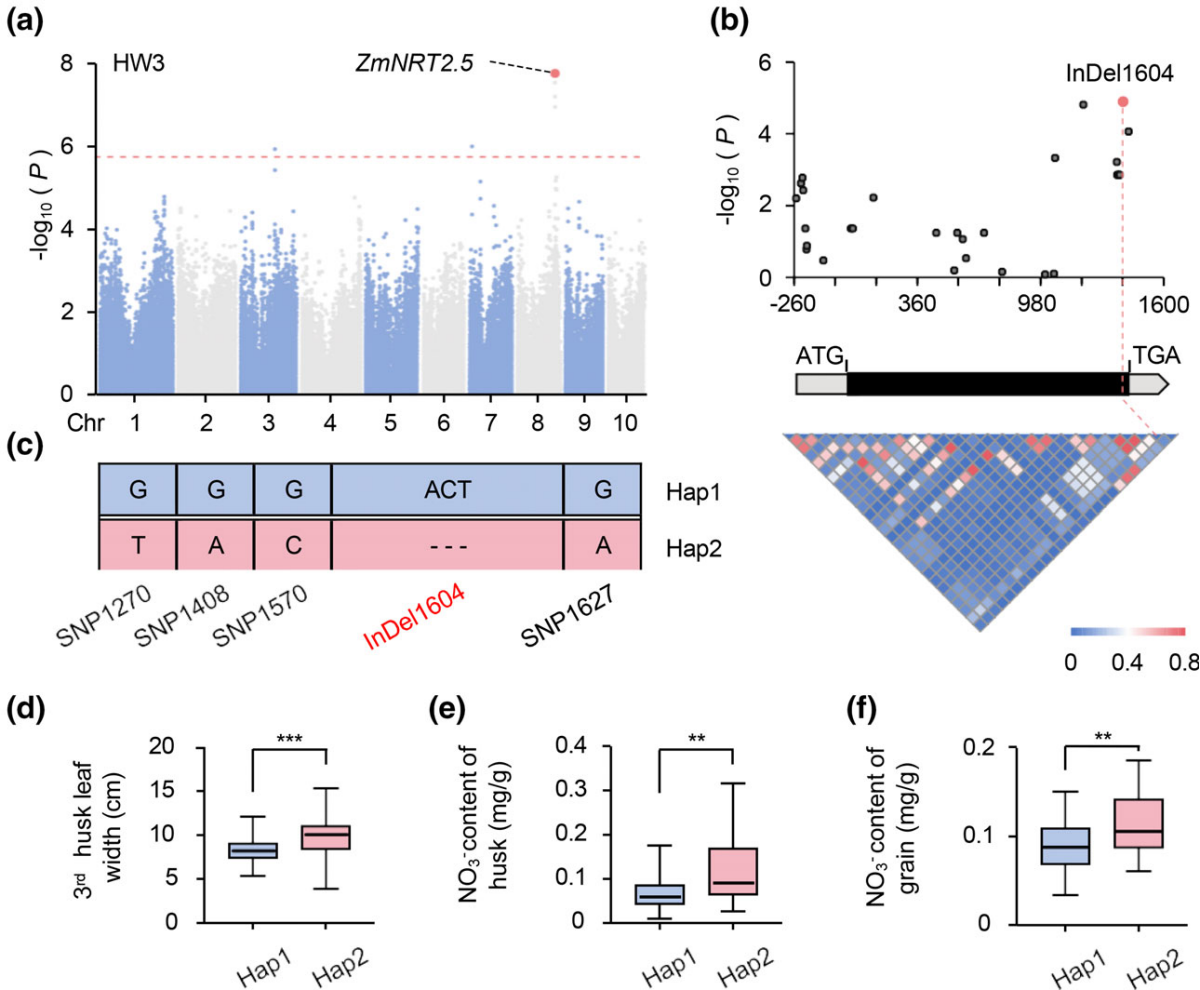
Natural variation in the coding sequence of *ZmNRT2.5* is correlated with maize husk leaf width. (a) Manhattan plot for the single‐nucleotide polymorphisms (SNPs) associated with the width of the third husk leaf. The dashed horizontal line indicates the Bonferroni‐adjusted significance threshold (*P* = 1.8 × 10^−6^). The red dot indicates the −log_10_(*P*) value for the SNP located within the candidate gene Zm00001d011679. HW3, width of third husk leaf. (b) *ZmNRT2.5*‐based association mapping and pairwise linkage disequilibrium (LD) analysis. A diagram of the *ZmNRT2.5* locus, including the 5′ and 3′ untranslated regions (grey boxes) and the single exon (black box), is shown. The most statistically significant insertion–deletion (InDel) is highlighted in red. (c) Haplotypes (Haps) of *ZmNRT2.5* among the maize natural variants according to InDel1604 and four other SNPs. (d) Width of the third husk leaf for each haplotype group (Hap1 *𝑛* = 76, Hap2 *𝑛* = 80; ***P = 1.66 × 10^−10^). (e, f) NO_3_^−^ content of husk leaf (e) (Hap1 *𝑛* = 29, Hap2 *𝑛* = 43; **P = 2.78 × 10^−3^) and grains (f) (Hap1 *𝑛* =26, Hap2 *𝑛* = 24; **P = 8.30 × 10^−3^) between randomly selected Hap1 and Hap2 inbred lines. *𝑛* indicates the number of genotypes belonging to each haplotype group. Statistical significance was determined using the Wilcoxon rank‐sum test. **P < 0.01, ***P < 0.001.

To investigate the genetic basis of husk leaf width variation in maize, we measured the width of the third husk leaf in a diversity panel of 508 maize inbred lines grown in Tieling (Liaoning, China) in 2017. To comprehensively evaluate the phenotypic variations in the population, we collected phenotypic data from plants grown in three environments, namely Tieling, Sanya and Beijing, to obtain a best linear unbiased prediction (BLUP). Using 556 944 SNPs (minor allele frequency [MAF] ≥ 0.05) covering the entire maize genome, we performed a GWAS to identify the loci underlying variation in maize husk leaf width. When employing a standard mixed linear model (MLM), we identified 55 SNPs as being significantly associated with the width of the third maize husk leaf (Figure 1a). The peak SNP (Chr8_158417048, *P* = 1.67 × 10^−8^) was located in the coding sequence (CDS) of Zm00001d011679, encoding a high‐affinity nitrate transporter (NRT) member (hereafter termed *ZmNRT2.5*, Figure S1 and Table S1).

To fully characterise the extent of genetic variation present in *ZmNRT2.5*, we resequenced the *ZmNRT2.5* gene from 156 maize inbred lines, covering its 1563‐bp intronless coding region, a 261‐bp 5′ untranslated region (5′ UTR) and a 176‐bp 3′ UTR, leading to the identification of 23 SNPs and 5 InDels (MAF ≥0.05; Table S2). An association analysis of these variants and the husk leaf width phenotype using TASSEL 5.0 revealed that a 3‐bp InDel (InDel1604), located at the 3′ end of the coding region, shows the highest association with maize husk leaf width, indicating that InDel1604 is likely the causal variant for the observed variation (Figure 1b). InDel1604 results in the presence or absence of one threonine residue in the C terminus of ZmNRT2.5. Based on the genotype for five polymorphisms present in the 3′ end of the gene, we classified the 156 maize inbred lines into two haplotype groups (Hap1 and Hap2), with Hap1 harbouring the 3‐bp insertion responsible for the addition of the threonine residue (Figure 1c). The Hap1 and Hap2 groups are composed of 76 and 80 inbred lines, respectively, with the Hap2 group exhibiting significantly wider husk leaves than the Hap1 group (*P* = 1.67 × 10^−10^; Figure 1d). As ZmNRT2.5 is predicted to be a nitrate transporter, we measured the nitrate (NO_3_^−^) content of husk leaves and grains from Hap1 and Hap2 inbred lines (Table S4). The NO_3_^−^ contents of husk leaves and grains from Hap2 inbred lines were significantly higher than those of Hap1 inbred lines (Figure 1e,f). These results indicate that the 3‐bp InDel in *ZmNRT2.5* affects the width of maize husk leaves and the NO_3_^−^ contents of husk leaves and grains in a natural maize population.

### A potential ZmNRT2.5–ZmNPF5 interactive module for maize husk leaf width regulation

**Figure 2:**
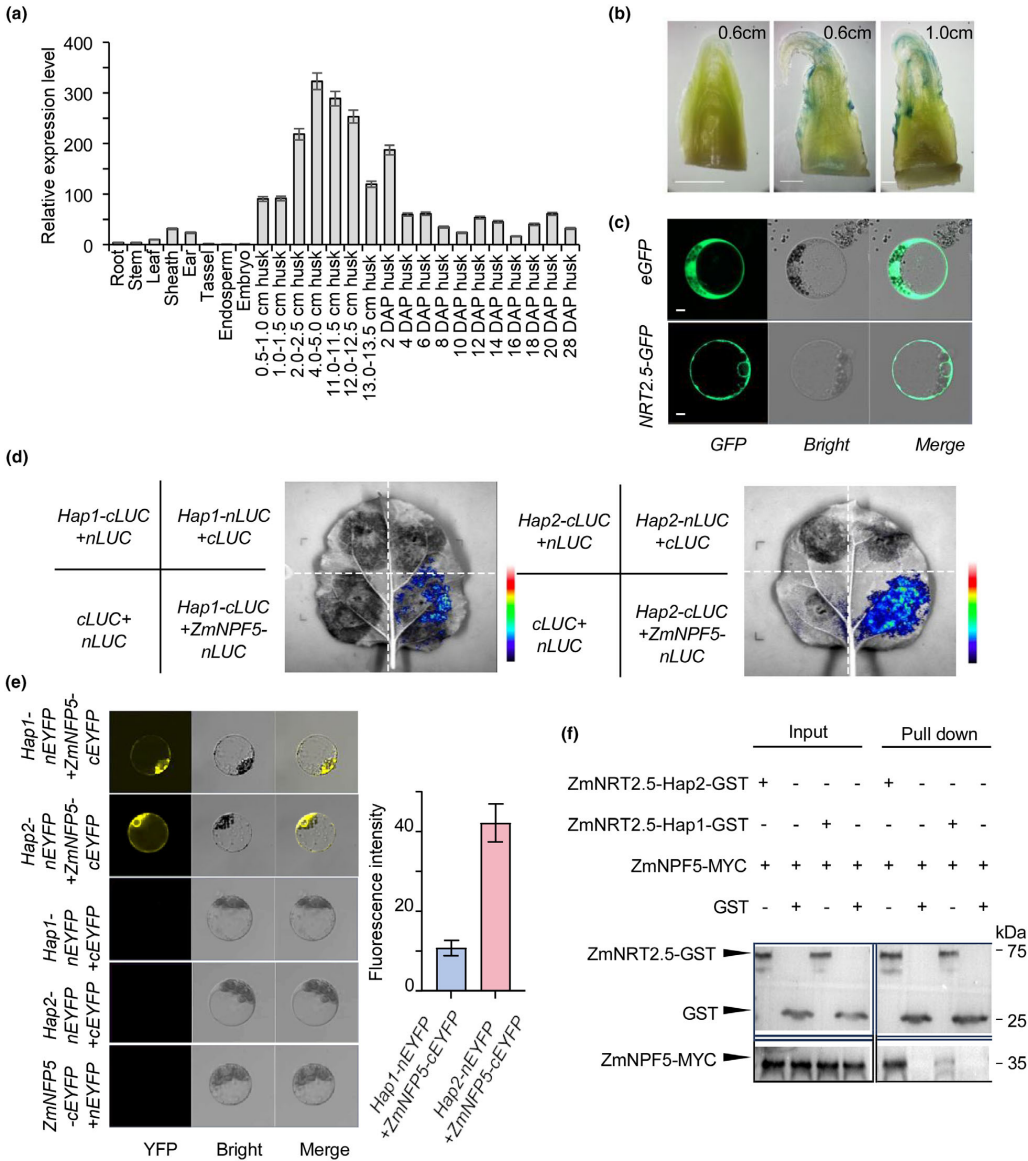
ZmNRT2.5 is associated with ZmNPF5. (a) Tissue expression pattern of *ZmNRT2.5*, determined by RT‐qPCR. (b) GUS staining of ears at different stages from *ZmNRT2.5pro:GUS* transgenic plants. Scale bars, 5 mm. (c) Subcellular localisation of ZmNRT2.5‐GFP in maize protoplasts. Bright, brightfield. Scale bars, 5 μm. (d) Split firefly luciferase complementation assay in *N. benthamiana* leaves showing the interaction between the Hap1 and Hap2 variants of ZmNRT2.5 and ZmNPF5. (e) Bimolecular fluorescence complementation (BiFC) assay in maize protoplasts confirming the interaction between the Hap1 and Hap2 variants of ZmNRT2.5 and ZmNPF5. Bright, brightfield. Scale bars, 5 μm. Fluorescence intensity detected in *Hap1‐nEYFP + ZmNFP5‐cEYFP* and *Hap2‐nEYFP + ZmNFP5‐cEYFP* was quantified using ImageJ software. (f) *In vitro* pull‐down assay showing the interaction between ZmNRT2.5 and ZmNPF5. Recombinant GST and ZmNRT2.5‐GST proteins were purified using Glutathione beads and incubated with ZmNPF5‐MYC protein, respectively.

To explore the role of ZmNRT2.5 in the regulation of husk leaf development, we investigated the expression pattern of *ZmNRT2.5*, noting the high expression of this gene in the early stage of maize husk leaf development (Figure 2a). This result suggested that ZmNRT2.5 may regulate husk leaf development at an early stage. Examination of *ZmNRT2.5pro*:*GUS* transgenic plants harbouring a transgene consisting of the *ZmNRT2.5* promoter driving the expression of the *β‐glucuronidase* (*GUS*) reporter mainly detected GUS activity in developing husk leaves (Figure 2b), but not in stem or male tissues (Figure S2a). We also analysed the subcellular localisation of ZmNRT2.5 by transfecting a *ZmNRT2.5‐GFP* construct (encoding a fusion of ZmNRT2.5 to the green fluorescent protein [GFP]) in maize protoplasts and infiltrating the same construct into *Nicotiana benthamiana* leaves via Agrobacterium‐mediated infiltration (Figures 2c and S2b). We detected fluorescent signals from ZmNRT2.5‐GFP at the plasma membrane in both cell types.

As transcriptional changes may contribute to the variation in *ZmNRT2.5*, we measured *ZmNRT2.5* transcript levels in randomly selected Hap1 (*n* = 10) and Hap2 (*n* = 10) inbred lines by reverse‐transcription quantitative PCR (RT‐qPCR). The *ZmNRT2.5* mRNA levels were comparable in Hap1 and Hap2 lines (Student *t*‐test, *P* = 0.54; Figure S2c), suggesting that the observed variation in *ZmNRT2.5* is not associated with changes in the transcriptional output from this locus. A phylogenetic tree for ZmNRT2.5 and related proteins showed that ZmNRT2.5 and AtNRT2.5 belong to the same major cluster (Figure S1). A multiple protein sequence alignment between proteins from dicotyledonous and monocotyledonous species revealed that most proteins are Hap2‐type (lacking a Thr residue), with a few being Hap1‐type (with the Thr insertion) (Figure S3a). Like all members of the NRT family, ZmNRT2.5 contains 10 putative transmembrane domains and a long hydrophilic chain in its C terminus (Figure S3b). To assess the potential influence of the presence/absence of the threonine residue on protein structure, we submitted the ZmNRT2.5^Hap1^ and ZmNRT2.5^Hap2^ protein sequences to a prediction website (https://harrier.nagahama‐i‐bio.ac.jp/sosui). This analysis suggested that the presence or absence of the threonine in the C terminus, which primarily functions as post‐translational modifications and participates in protein‐protein interactions, may lead to changes in hydrophilicity and net charge (Figure S3c). Previous studies have reported that NRT2 family members interact with NRT3.1 in Arabidopsis (Jacquot *et al*., 2020; Kotur *et al*., 2012; Okamoto *et al*., 2006), and that phosphorylation of Thr‐521 is highly conserved and crucial for nitrate transport activity (Jacquot *et al*., 2020). RT‐qPCR analysis revealed that *ZmNPF5* is highly expressed in the husk leaf, as was *ZmNRT2.5* (Figure S3d), raising the possibility that ZmNRT2.5 and ZmNPF5 may interact for the regulation of husk leaf width. Accordingly, we investigated the interaction of ZmNRT2.5^Hap1^ and ZmNRT2.5^Hap2^ with ZmNPF5 by conducting a split‐luciferase complementation assay in *N. benthamiana* leaves. Indeed, we detected luminescence from reconstituted firefly luciferase (LUC), indicating that ZmNPF5 does interact with both ZmNRT2.5 variants, with a stronger LUC activity detected for ZmNPF5 and ZmNRT2.5^Hap2^ than for ZmNPF5 and ZmNRT2.5^Hap1^ (Figure 2d). We further confirmed the interaction of ZmNRT2.5 and ZmNPF5 in a bimolecular fluorescence complementation (BiFC) assay using constructs encoding ZmNRT2.5^Hap1^ or ZmNRT2.5^Hap2^ fused to the N‐terminal half of the enhanced yellow fluorescent protein (nEYFP) and ZmNPF5 fused to the C‐terminal half of enhanced YFP (cEYFP) transfected into maize protoplasts. We detected YFP fluorescence in both sets of protoplasts and protoplasts co‐transfected with *ZmNRT2.5* ^*Hap2*^ *‐nEYFP* and *ZmNPF5‐cEYFP* exhibited stronger fluorescence intensity than those co‐transfected with *ZmNRT2.5* ^*Hap1*^ *‐nEYFP* and *ZmNPF5‐cEYFP* (Figure 2e). The *in vitro* pull‐down assay in N. benthamiana leaves was conducted, and the results revealed that the concentration of ZmNPF5‐MYC protein was higher in the sample containing ZmNRT2.5‐Hap2‐GST than in the sample containing ZmNRT2.5‐Hap1‐GST (Figures 2f and S4a,b). These results indicate that ZmNRT2.5 interacts with ZmNPF5, which may contribute to the regulation of maize husk leaf width.

To explore the role of interaction protein *ZmNPF5* in regulating maize husk width, we performed a GWAS analysis to identify the loci underlying variation in *ZmNPF5* with maize husk width. Using 556 944 SNPs covering the entire maize genome, we analysed the natural variation in the 500‐bp promoter and genomic region of *ZmNPF5*, and a SNP (SNP‐542) located at the promoter of *ZmNPF5* showed the highest association with maize husk leaf width (Figure S5a). Based on the SNP‐542, we classified the maize inbred lines into two haplotype groups: NPF5^Hap1^ and NPF5^Hap2^. The NPF5^Hap2^ group exhibited significantly wider husk leaves than the NPF5^Hap1^ group (*P* = 2.0 × 10^−4^; Figure S5b). Furthermore, based on the InDel1604 of ZmNRT2.5 and the SNP‐542 of ZmNPF5, the maize inbred lines were further classified into four haplotypes: NRT2.5^Hap1^/NPF5^Hap1^, NRT2.5^Hap1^/NPF5^Hap2^, NRT2.5^Hap2^/NPF5^Hap1^ and NRT2.5^Hap2^/NPF5^Hap2^. Statistically, the husk leaf width of NRT2.5^Hap2^/NPF5^Hap2^ inbred lines (*n* = 116) was significantly wider than that of NRT2.5^Hap1^/NPF5^Hap1^ lines (*n* = 26; *P* = 2.8 × 10^−6^; Figure S5c). Therefore, *ZmNRT2.5*, in conjunction with *ZmNPF5*, is involved in regulating maize husk width.

### *ZmNRT2.5* positively regulates maize husk leaf width

**Figure 3:**
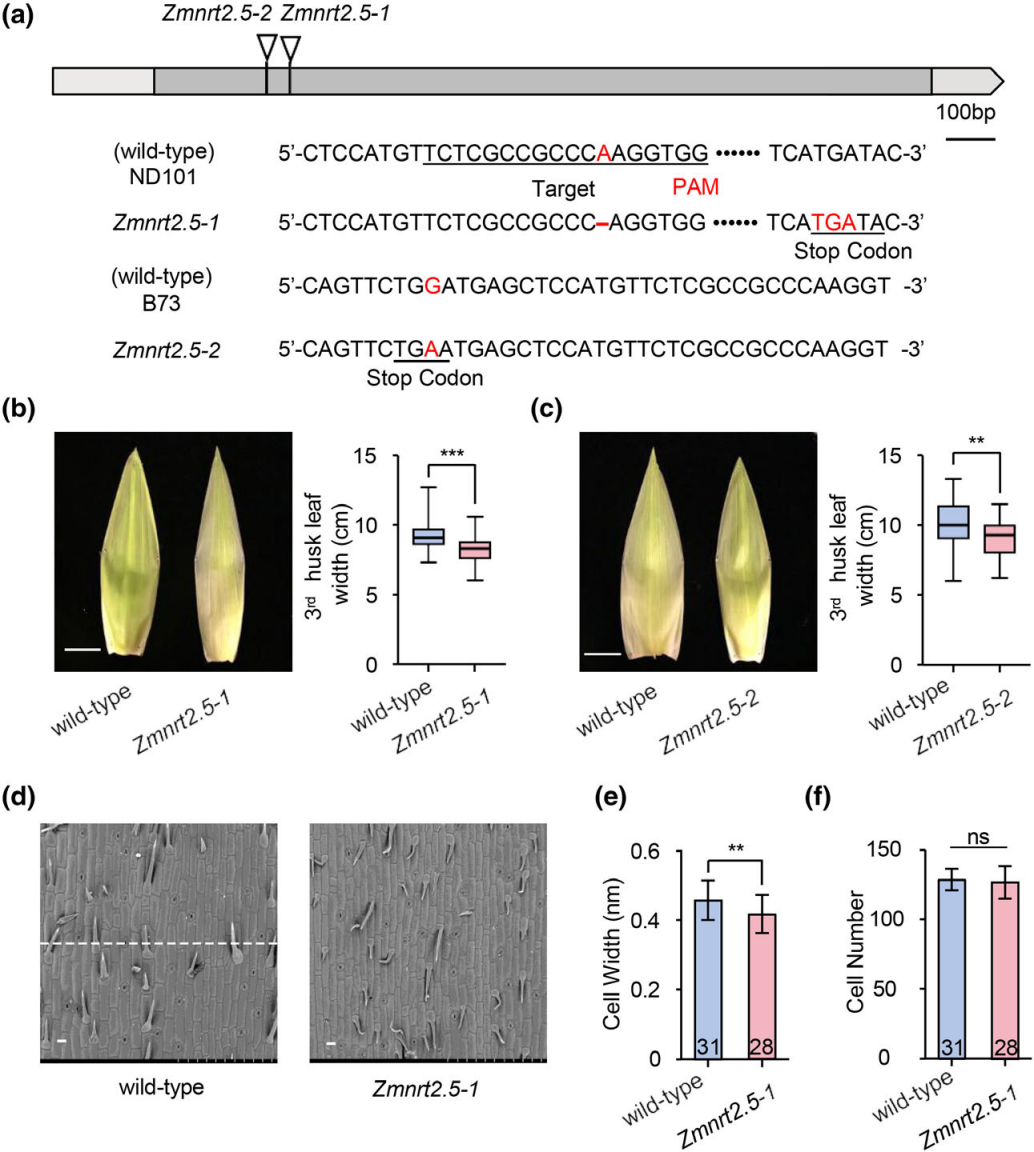
ZmNRT2.5 positively regulates maize husk leaf width. (a) Gene structure of the *ZmNRT2.5* locus with the position of mutations in two mutant alleles. *Zmnrt2.5‐1* was generated by CRISPR/Cas9‐mediated editing, harbouring a 1‐bp deletion in the *ZmNRT2.5* coding sequence causing the introduction of a premature stop codon. The EMS mutant *Zmnrt2.5‐2* carries a 1‐bp substitution from G to A in the coding sequence, introducing a premature stop codon. The deletion and stop codon are highlighted in red. (b) Left, representative photograph of the third husk leaf of the *Zmnrt2.5‐1* mutant and its corresponding wild‐type (WT, ND101) plants. Scale bar, 100 mm. Right, width of the third husk leaf from WT (ND101) and the *Zmnrt2.5‐1* mutant. ND101 *𝑛* = 53, *Zmnrt2.5‐1 𝑛* = 74; ***P < 0.001. (c) Left, representative photograph of the third husk leaf of the *Zmnrt2.5–2* mutant and its corresponding WT (B73) plants. Scale bar, 100 mm. Right, width of the third husk leaf from WT (B73) and the *Zmnrt2.5‐2* mutant. B73 *𝑛* = 40, *Zmnrt2.5‐1 𝑛* = 54; **P < 0.01. *𝑛* denotes the number of independent plants assessed. Statistical significance was determined using the Wilcoxon rank‐sum test. **P < 0.01, ***P < 0.001. (d) Scanning electron microscopy (SEM) images of the third husk leaf (from outer to inner layer) at 20 days after pollination (DAP) in WT (ND101) and *Zmnrt2.5‐1* plants. Scale bars, 1 mm. The white dashed line indicates the measured cells in the middle region of the SEM image. (e, f) Width (e) and number (f) of abaxial epidermal cells in WT (ND101) and *Zmnrt2.5‐1* plants. The number of epidermal cells was calculated by dividing the average husk leaf width by the average width of husk leaf epidermal cells. The numbers in the bars indicate the number of plants assessed for each genotype. For each plant, 10 regions in the middle section of the third husk leaf were selected for SEM analysis. Data are presented as means ± SD; statistical significance was determined using the Student's *t*‐test. ns, not significant, **P < 0.01.

The GWAS and association analysis above suggested *ZmNRT2.5* as a candidate gene that affects maize husk leaf width. To test this hypothesis, we generated a mutant of *ZmNRT2.5* using CRISPR/Cas9‐mediated gene editing in the inbred line ND101 (Xing *et al*., 2014). We identified one mutant line, referred to as *Zmnrt2.5–1*, with a 1‐bp deletion near the beginning of the coding region, resulting in the introduction of a premature stop codon and leading to a truncated ZmNRT2.5 protein (Figure 3a). In addition, we identified one ethyl methane sulfonate (EMS) mutant (*Zmnrt2.5‐2*; ems4‐9bc4f) with a premature stop codon in the inbred line B73 from a maize EMS mutant library (Lu *et al*., 2018) (Figure 3a). The *Zmnrt2.5‐1* and *Zmnrt2.5‐2* mutants were backcrossed to their respective wild‐type background line for three generations. Phenotypic analysis revealed that the *Zmnrt2.5* null alleles produced significantly thinner maize third husk leaves (Figure 3b,c, Tables S5 and S6) but showed no significant effect on any other agronomic trait tested, such as plant height, ear height, third husk leaf length, husk leaf number, ear leaf length, ear leaf width, sheath length and sheath width (Figures S6 and S7). Furthermore, we generated two independent transgenic overexpression (OE) maize lines (OE#1 and OE#2). RT‐qPCR analysis demonstrated a substantial increase in *ZmNRT2.5* expression levels compared with the wild type (Figure S8a). Significantly wider husk leaves were observed in OE#1 and OE#2 plants than in control plants (Figure S8b). The husk leaf length was comparable between OE#1, OE#2, and wild‐type plants (Figure S8c). Together, these results indicate that *ZmNRT2.5* is a positive regulator of husk leaf width in maize.

Leaf size is determined by both cell division and cell expansion (Hareven *et al*., 1996; Janssen *et al*., 1998; Tsuge *et al*., 1996). To uncover the cellular mechanism behind the change in husk leaf width, we used scanning electron microscopy (SEM) to measure the width and number of epidermal cells along the husk leaf‐width axis of the third husk leaf in ND101 and the *Zmnrt2.5‐1* mutant (Figure 3d). Although we counted comparable cell numbers in the husk leaves of *Zmnrt2.5‐1* and wild‐type plants, *Zmnrt2.5‐1* plants did show significantly narrower epidermal cells (Figure 3e,f). These results indicate that *ZmNRT2.5* affects husk leaf width by modulating cell expansion, but not cell division.

Our previous study showed that loss of function of *RHW1* led to narrower maize husk leaves (Xia *et al*., 2023). To explore the genetic relevance of *ZmNRT2.5* and *RHW1*, we generated the *Zmnrt2.5‐1 rhw1‐1* double mutant by genetic crossing; the double mutant had significantly narrower maize third husk leaves than the *Zmnrt2.5‐1* and *rhw1* single mutants (Figure S8d,e). Thus, we conclude that *ZmNRT2.5* and *RHW1* regulate the width of maize husk leaves via two distinct pathways.

### *ZmNRT2.5* transports nitrate from husk leaves to grains

**Figure 4:**
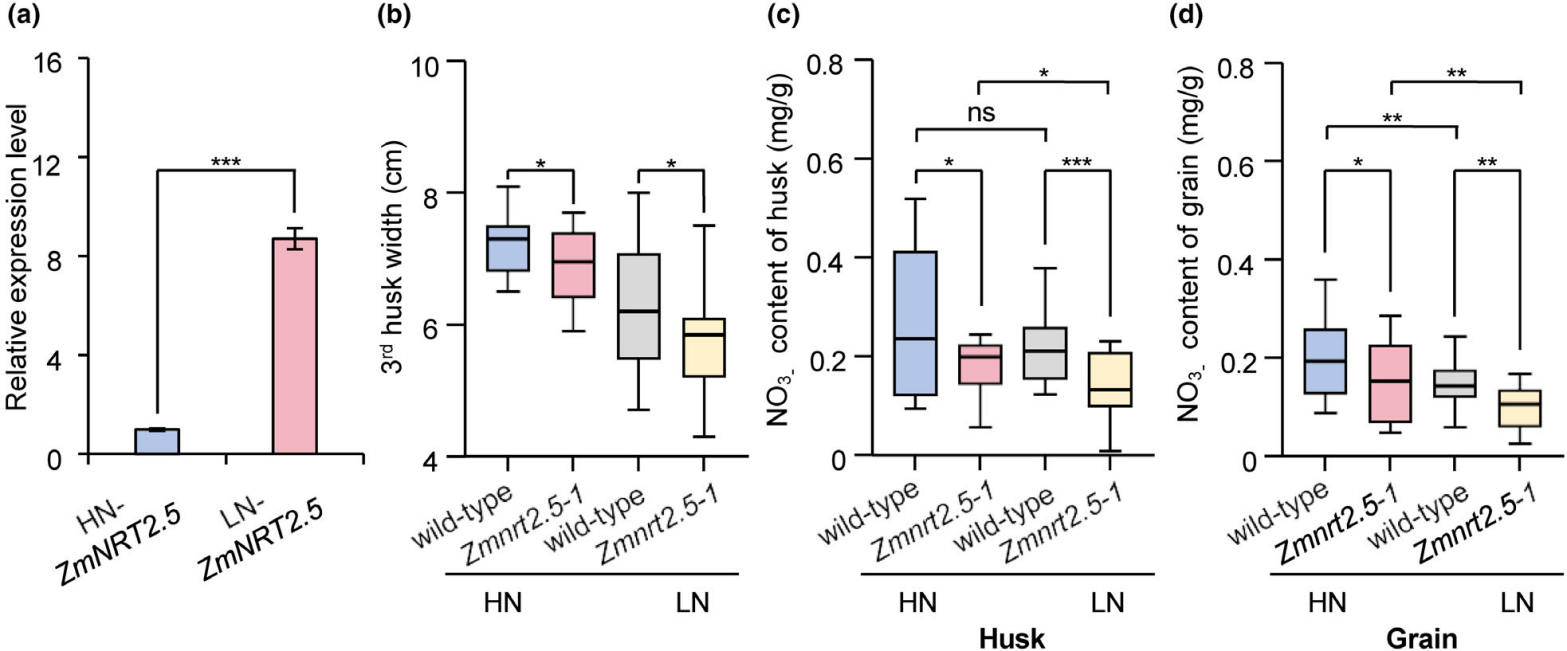
The NO_3_^−^ content of husk leaves and grains in maize grown under high‐nitrogen or low‐nitrogen conditions. (a) RT‐qPCR analysis of *ZmNRT2.5* expression under high‐nitrogen (HN) and low‐nitrogen (LN) conditions was performed using wild‐type husks at 20 DAP. Data are presented as means ± SD from three independent biological replicates. 1 kg N/100 m^2^ for the low‐nitrogen condition and 2 kg N/100 m^2^ for the high‐nitrogen condition. (b) Width of the third husk leaf at 20 DAP in the WT (ND101) and *Zmnrt2.5‐1* mutant under HN and LN conditions. *n* = 20, 26, 34, and 32, respectively. (c) NO_3_^−^ content of husk leaves from WT (ND101) and the *Zmnrt2.5‐1* mutant grown under HN or LN conditions. *n* = 20, 18, 22 and 20, respectively. (d) NO_3_^−^ content of grains from WT (ND101) and the *Zmnrt2.5‐1* mutant grown under HN or LN conditions. *n* = 20, 18, 22 and 20, respectively. All husk leaves and grains were collected at 20 DAP. Statistical significance was determined using the Wilcoxon rank‐sum test. ns, not significant, *P < 0.05, **P < 0.01, ***P < 0.001.

*ZmNRT2.5* is a member of a potential high‐affinity NO_3_^−^ transport system (HATS); *NRT2.5* expression is induced upon nitrate limitation, a response that is conserved in monocots and dicots (Lezhneva *et al*., 2014). To investigate the coordinated utilisation of NO_3_^−^, we cultivated the *Zmnrt2.5‐1* mutant and its wild type (ND101) under varying nitrate supply conditions in field experiments at Zhuozhou (Hebei, China). RT‐qPCR analysis revealed that *ZmNRT2.5* expression is highly induced under low‐nitrogen (LN) conditions compared to high‐nitrogen (HN) conditions (Figure 4a). The width of maize husk leaves was significantly narrower in the *Zmnrt2.5‐1* mutant grown under both HN and LN conditions than in the wild type grown under the same conditions (Figure 4b). Furthermore, husk leaf width was significantly narrower in plants grown under the LN condition than those grown under the HN condition (Figure S9a). We also measured the content of NO_3_^−^ in husk leaves and grains from the wild‐type and the *Zmnrt2.5‐1* mutant under LN and HN conditions. Compared to the wild type, we observed a significant decrease in the NO_3_^−^ content of maize husk leaves from plants grown under both HN and LN conditions, with a significantly lower NO_3_^−^ content for husk leaves of plants grown under the LN condition relative to that under the HN condition (Figures 4c and S9b). Consistent with the results observed in husk leaves, the NO_3_^−^ content of *Zmnrt2.5‐1* grains was significantly lower than that of the wild type when grown under either the HN or LN condition (Figures 4d and S9c). Similarly, we observed a significant increase in NO_3_^−^ content in the maize grains of OE#1 and OE#2 compared to their wild type (Figure S9d). These observations suggest that *ZmNRT2.5* contributes to the transport of nitrate from the husk leaf to the grain in the field and plays an important role in nitrate transport under low‐nitrogen conditions.

### *ZmNRT2.5* regulates the expression of nitrate transport‐related genes

**Figure 5:**
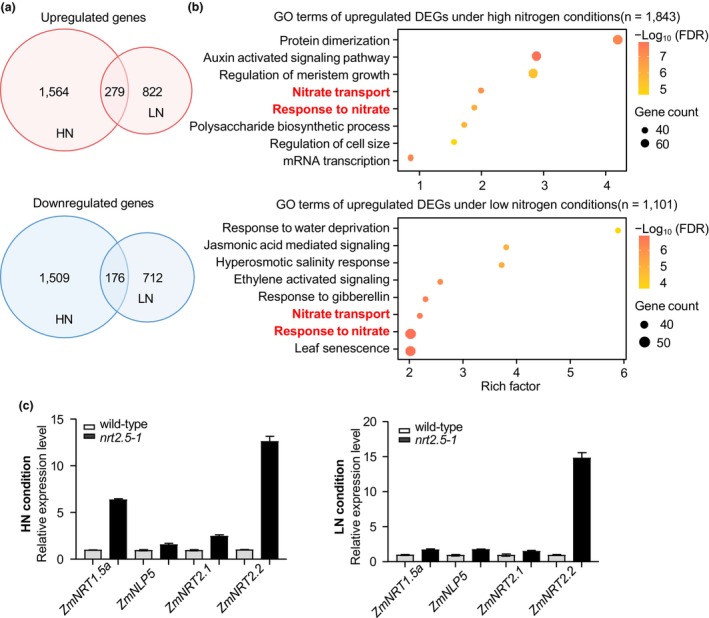
Transcriptome analysis reveals changes in the expression of nitrate transport–related genes under high‐nitrogen and low‐nitrogen conditions. (a) Venn diagrams showing the number of differentially expressed genes (DEGs) in wild‐type (WT) and the *Zmnrt2.5–1* mutant grown under high‐nitrogen (HN) or low‐nitrogen (LN) conditions. 1 kg N/100 m^2^ for the low‐nitrogen condition and 2 kg N/100 m^2^ for the high‐nitrogen condition for the entire growth period. (b) Gene ontology (GO) term enrichment analysis of upregulated DEGs in *Zmnrt2.5‐1* relative to the WT under HN (upper panel) or LN conditions (lower panel). The most significantly enriched GO terms are shown. Circle size indicates the number of DEGs, and the colour gradient indicates the significance of enrichment. (c) RT‐qPCR confirmation of the response to nitrate and nitrate transport in wild‐type and *Zmnrt2.5‐1* husks under HN and LN conditions.

To evaluate the effect of the loss of ZmNRT2.5 function on gene expression in husk leaves, we performed RNA sequencing (RNA‐seq) of ears (about 5.0 cm in length) from wild‐type (ND101) plants and the *Zmnrt2.5‐1* mutant at the V12 (12th vegetative leaf) stage. We identified 1685 downregulated genes and 1843 upregulated genes in the *Zmnrt2.5‐1* mutant compared to the wild type when grown under the HN condition (Figures 5a, S10a and Table S7). Under the LN condition, there were 888 downregulated genes and 1101 upregulated genes in the mutant compared to the wild type (Figures 5a, S10b and Table S8). A gene ontology (GO) term enrichment analysis performed on the differentially expressed genes (DEGs) under the HN or LN condition revealed a high enrichment for genes related to nitrate transport among the upregulated genes (Figure 5b). We validated the expression patterns of several genes involved in nitrate response and transport by RT‐qPCR (Figure 5c). Together, these results indicate that the dysfunction of ZmNRT2.5 may alter the transcript levels of genes involved in nitrate response and cell morphogenesis, which would contribute to the transport of nitrate and cell morphology in maize husk leaves.

### Potential of *ZmNRT2.5* in maize breeding

**Figure 6:**
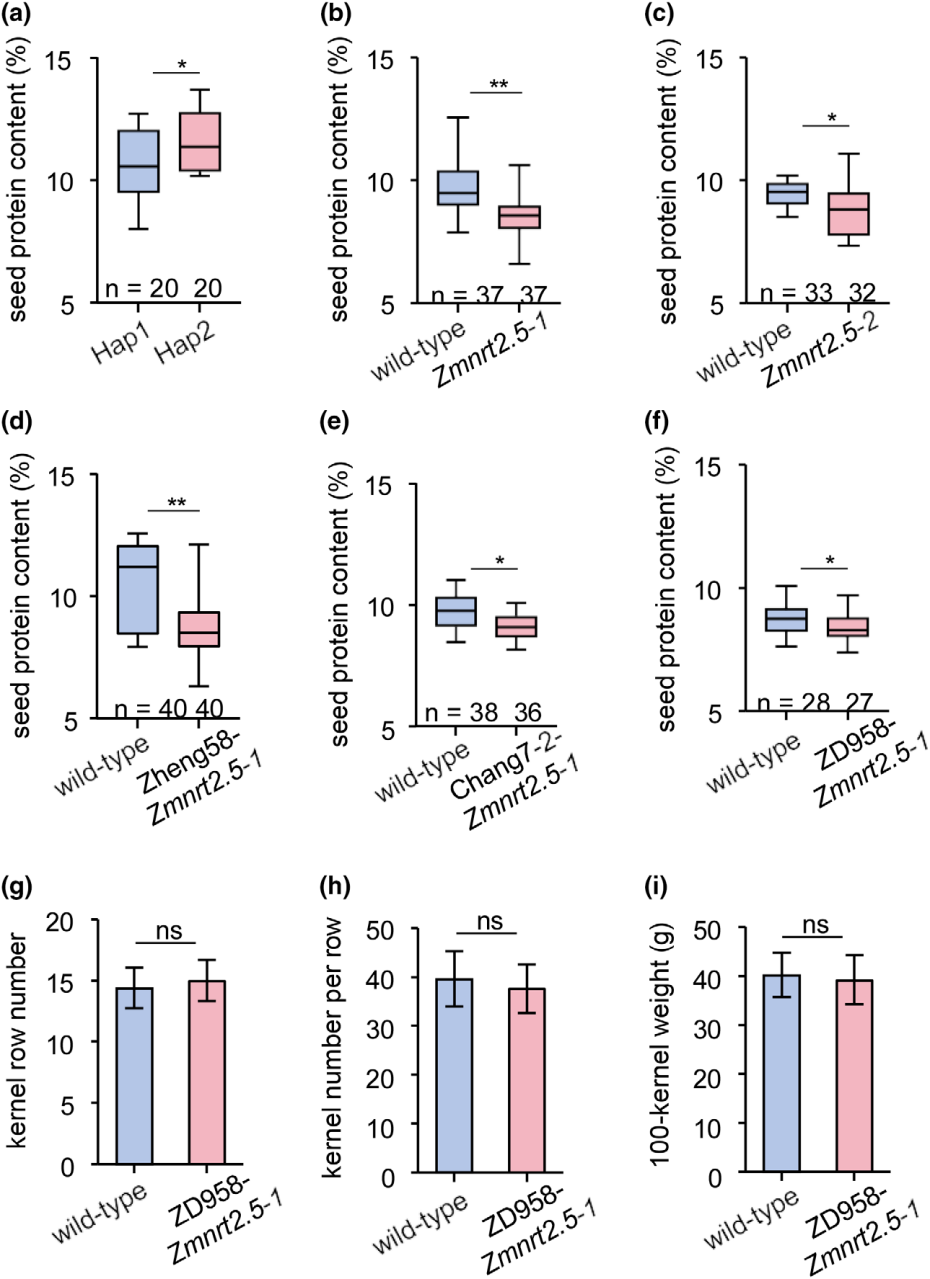
Seed protein content and grain yield in maize with different ZmNRT2.5 genotypes. (a–c) Seed protein content of inbred maize lines with the Hap1 or Hap2 haplotype of *ZmNRT2.5* (a), *Zmnrt2.5‐1* and its wild type (ND101) (b), and *Zmnrt2.5‐2* and its wild type (B73) (c) Statistical significance was determined using the Wilcoxon rank‐sum test. *P < 0.05, **P < 0.01. (d–f) Seed protein content of the introgressed knockout lines Zheng58‐*Zmnrt2.5‐1* (d), Chang7‐2‐*Zmnrt2.5‐1* (e) and ZD958‐*Zmnrt2.5‐1* (f). Statistical significance was determined using the Wilcoxon rank‐sum test. *P < 0.05, **P < 0.01. (g–i) Kernel row number (g), kernel number per row (h) and hundred‐kernel weight (i) of WT (ZD958) and ZD958‐*Zmnrt2.5‐1*. *n* = 100 respectively. Data are presented as means ± SD; Statistical significance was determined using the Student's *t*‐test. ns, not significant.

As *ZmNRT2.5* affected nitrate transport from husk leaves to grains, we asked whether *ZmNRT2.5* might contribute to the protein content of seeds in maize hybrids. To this end, we randomly selected 20 Hap1 and 20 Hap2 inbred lines and planted them at the Tieling site. The protein content of seeds from the Hap2 inbred lines (11.4%) was 5.7% higher than that of seeds from the Hap1 inbred lines (10.8%) (Figure 6a and Table S9). The protein content of *Zmnrt2.5‐1* and *Zmnrt2.5‐2* mutant seeds was significantly lower than that of the respective wild types (8.5% for *Zmnrt2.5‐1* versus 9.6% for ND101, 8.4% for *Zmnrt2.5‐2* versus 9.4% for B73; Figure 6b,c and Table S10). In comparison, there is no significant change in the protein content of *rhw1‐1* mutant seeds (Figure S11a). We also introgressed *Zmnrt2.5‐1* into Zheng58 (the maternal donor) and Chang7‐2 (the paternal donor) – two elite inbred lines that form the Zhengdan 958 (ZD958) hybrid, the most widely cultivated maize variety in China. ZD958 is also a nitrogen‐efficient maize hybrid at the seedling stage (Han *et al*., 2015). We crossed the Zheng58‐*Zmnrt2.5‐1* and Chang7‐2‐*Zmnrt2.5‐1* introgressed lines to generate a ZD958 hybrid with loss of ZmNRT2.5 function (designated ZD958*‐Zmnrt2.5‐1*), which we planted alongside its wild‐type hybrid control ZD958 in Tieling in 2023. The seed protein content of the two introgression lines (Zheng58*‐Zmnrt2.5‐1* and Chang7‐2*‐Zmnrt2.5‐1*) decreased significantly compared to their respective wild types (Figure 6d,e). Similarly, ZD958*‐Zmnrt2.5‐1* seeds had a protein content of 8.1%, corresponding to an 8.05% decrease compared to ZD958 seeds (8.8%) (Figure 6f and Table S11).

To assess whether the alteration in seed protein content caused by loss of ZmNRT2.5 function affects grain yield, we measured three traits directly related to grain yield, namely kernel row number, kernel number per row and hundred‐kernel weight, in a field trial at Tieling. Compared to their corresponding wild‐type ZD958, ZD958*‐Zmnrt2.5‐1* plants showed no significant difference in these three grain yield traits or in the other agronomic traits, including husk leaf number, ear length, ear diameter, ear weight, cob diameter and cob weight (Figures 6g–i and S11c–h), apart from the significant difference in 3rd husk leaf width (Figure S11b,i). Meanwhile, the protein content of *ZmNRT2.5* OE#1 and OE#2 seeds was significantly higher than that of wild types (9.6% for OE#1 and 9.7% for OE#2 versus 8.9% for ND101; Figure S11j). These results suggest that *ZmNRT2.5* has the potential to improve the protein content of maize seeds through plant breeding without negatively affecting grain yield.

## Discussion

The maize husk leaf, a modified leaf surrounding the ear, contributes substantially to grain filling and to protecting the ears from pest damage and pathogen infection (Dean *et al*., 1986; Demissie *et al*., 2008; Warfield and Davis, 1996). Notably, until now almost nothing was known about the gene regulatory network that specifically modulates husk leaf growth and kernel nutrient quality. In this study, we performed a GWAS for maize husk leaf width that identified a 3‐bp InDel within the 3′ end of the *ZmNRT2.5* coding region. This polymorphism affected the interaction strength of the putative nitrate transporters ZmNRT2.5 and ZmNPF5. Moreover, we showed that ZmNRT2.5 facilitates nitrate transport from husk leaves to grains under low‐nitrogen growth conditions, leading to an increase in maize seed protein content.

### *ZmNRT2.5* is involved in regulating husk leaf width

The Arabidopsis and maize genomes both encode seven NRT2 members, some *NRT*s being exclusively expressed in specific tissues or developmental stages (Jia *et al*., 2023). Although much work has been carried out on the *NRT* gene families of Arabidopsis and rice, far fewer studies have focused on their maize counterpart. Although members of the *NRT* transporter gene family are typically highly expressed in roots, the expression of *ZmNRT2.5* is higher in husk leaves than that in roots, and the homologous gene *TaNRT2.5* in wheat (*Triticum aestivum*) is highly expressed in the embryo and plays a crucial role in the accumulation of nitrate in seeds (Li *et al*., 2019). In this study, we demonstrated that *ZmNRT2.5* is involved in regulating husk leaf width in maize, based on four lines of evidence. First, *ZmNRT2.5* is highly and specifically expressed in maize husk leaves. Second, loss of function of *ZmNRT2.5* resulted in narrower husk leaves, which was observed in two independent homozygous *Zmnrt2.5* mutants compared to their corresponding wild‐type plants. Third, the cell number of husk leaves, as determined by SEM, was comparable between each mutant and their corresponding wild‐type plants, whereas the width of cells from husk leaves was significantly smaller in the two mutant backgrounds than in their respective wild types. Fourth, more importantly, the InDel in the 3′ end of *ZmNRT2.5* resulted in the presence or absence of a threonine residue in the C terminus of the protein, affecting the interaction strength of ZmNRT2.5 and ZmNPF5, thereby resulting in differential NO_3_^−^ content of husk leaves and husk leaf width between inbred lines harbouring one or the other haplotype. However, whether this natural variation affects the supply of NO_3_^−^ to husk leaf cells, which in turn influences cell growth and leads to the overall narrowing of husk leaves, will need to be explored.

### A 3‐bp InDel in the 3′ end of the *ZmNRT2.5* coding region affects the interaction strength of ZmNRT2.5 and ZmNPF5

Previous research indicated that a SNP (+2163G>A) in the coding region of *TILLER NUMBER 1* (*TN1*), causing an amino acid change (Arg‐83 to Gln‐83) in the bromo‐adjacent homology (BAH) domain, affects the interaction of TN1 with TN1 INTERACTION FACTOR 1 (TIF1) and negatively regulates tiller number in rice (Zhang *et al*., 2023). Moreover, a SNP (+731C>T) in the coding sequence of *TaNAM‐A1* affected the protein‐protein interaction between TaNAM‐A1 and TaNAC016‐3A, thereby affecting the transactivation of *PHEOPHORBIDE A OXYGENASE 4B* (*TaPAO‐4B*) expression and chlorophyll degradation after anthesis in wheat (Zhou *et al*., 2024). A 3‐bp insertion in the coding region of *Teosinte branched 2* (*OsTb2*) increased the number of tillers in rice, likely by affecting OsTb2 protein activity or altering its secondary structure (Lyu *et al*., 2020). The activity of NRT2 transporters often depends on their interaction with ancillary proteins NPF5 (NAR2 or NRT3) and NRT2 (Kotur *et al*., 2012). In this study, we found that the functional polymorphism in the *ZmNRT2.5* coding region affects the protein‐protein interaction strength between ZmNRT2.5 and ZmNPF5. This 3‐bp InDel results in the presence or absence of a Thr residue that affects the interaction intensity between ZmNRT2.5 and ZmNPF5, thereby altering husk leaf width and NO_3_^−^ content in husk leaves and grains. The C terminus of nitrate transporters is the main domain through which they bind to their ancillary proteins. All Arabidopsis NRT2 transporters, except for NRT2.7, can interact with NRT3.1 in yeast (Vidal *et al*., 2020). Furthermore, using *in silico* predictions, we revealed that the InDel polymorphism might alter the 3D structure of ZmNRT2.5, which may affect its function. In addition, the hydrophilicity of the protein contributes to its stability and facilitates its binding to other proteins (Herrwerth *et al*., 2003; Khmelnitsky *et al*., 1991). Therefore, from the perspective of the protein structure, whether the presence/absence of the threonine residue alters the binding strength of ZmNRT2.5 to ZmNPF5 by affecting its hydrophobicity needs experimental validation.

### Potential use of *ZmNRT2.5* for increasing seed protein content in breeding

ZmNRT2.5, a member of the NRT2 family, is part of a potential high‐affinity NO_3_^−^ transport system (HATS) (Jia *et al*., 2023). The husk leaf is thought to act as a source of nutrients, while kernels function as a storage reservoir, with nitrogen and other nutrients being continuously transported to the kernels (Fujita *et al*., 1995). As nitrate is one of the main sources of nitrogen, it will ultimately be converted into protein and stored in kernels (Cantrell and Geadelmann, 1981; Ogunbosoye and Odedire, 2022). Compared to their respective wild types, we detected a significant decrease in kernel protein content in the two *Zmnrt2.5* mutants characterised in this study, as well as in the two parental lines for the ZD958 hybrid into which we introgressed the *Zmnrt2.5‐1* mutation and their resulting ZD958‐*Zmnrt2.5‐1* hybrid, with no change in grain yield. Furthermore, kernels from inbred lines harbouring the Hap2 haplotype of *ZmNRT2.5* showed significantly higher protein content than those with the Hap1 haplotype. Given that *ZmNRT2.5* is highly expressed in husk leaves, it is possible that this gene is involved in the regulation of NO_3_^−^ translocation from husk leaves to grains in maize, thereby conferring variability of seed protein content between these two maize haplotypes. Thus, the favourable *ZmNRT2.5* allele is a promising tool for developing maize varieties with higher seed protein content, and future breeding programs may wish to introgress this allele into the parental lines of high‐yielding maize hybrids to breed commercially viable varieties with increased seed protein content.

## Methods

### Maize husk leaf width measurement

The association panel comprised 508 diverse maize inbred lines, comprising 60 lines from the Germplasm Enhancement of Maize (GEM) project, 223 lines from the International Maize and Wheat Improvement Center (CIMMYT), and 225 lines from China, all of which are publicly available on MaizeGO (http://www.maizego.org/). This maize panel can be classified into three distinct subpopulations: 27 Smooth Sheath (SS) lines, 70 Non‐Smooth Sheath (NSS) lines and 196 Tunicate Sheath (TST) lines, with the remaining 215 lines categorised as a mixed subpopulation. A field trial was carried out during the sowing season in Tieling, Liaoning province (42.1° N, 123.6° E) in 2017. Each inbred line was cultivated in a single‐row plot with two replicates following a complete randomised block design. Upon maturity, husk leaf width was assessed by measuring the middle section of the third husk leaf (counting from outside to inside). Phenotypic data were collected from three independent biological replicates, and the average of these three measurements was used to represent the trait for each inbred line.

### Genome‐wide association study (GWAS)

A total of 543 641 single‐nucleotide polymorphisms (SNPs) were selected, with a minor allele frequency (MAF) of at least 0.05 for GWAS by integrating data from two genotyping platforms: RNA‐seq and SNP arrays (Salvi *et al*., 2007). The best linear unbiased prediction (BLUP) was calculated for the phenotypic data collected at Tieling and two other environments (Hainan and Beijing; data from Cui et al., 2016). The lmer function in the R package lme4 was used to perform the BLUP analysis (Douglas *et al*., 2014). To analyse the association with husk leaf width, a mixed linear model (MLM) was employed incorporating both K and Q matrices to mitigate spurious associations, as implemented in the TASSEL V5.0 software package (Bradbury *et al*., 2007). We identified 95 742 markers in approximate linkage equilibrium with each other using PLINK (Purcell *et al*., 2007), setting the threshold of the linkage disequilibrium (LD) value *R* ^2^ to 0.2. Subsequently, uniform Bonferroni‐corrected thresholds were applied, setting α to 1 for MLM to establish significance cutoffs, in alignment with prior studies (Li *et al*., 2012; Mao *et al*., 2015; Yang *et al*., 2014). Consequently, suggestive *P*‐values were calculated using 1/*n* and 0.05/*n* (*n* = 95 742), yielding final significance *P*‐values of 1.67 × 10^−8^ for the MLM in this association study. Manhattan plots were generated using the R package CMplot. The phenotypic variation attributable to significant SNPs was estimated through an analysis of variance (ANOVA), following the methodology described by Wang *et al*. (2016).

### Analysis of *ZmNRT2.5*‐based association with husk leaf width

A total of 156 maize inbred lines were used to investigate the correlation between genetic variation in *ZmNRT2.5* and maize husk leaf width (Table S2). Based on the B73 reference genome sequence (B73 RefGen_v4) (Jiao *et al*., 2017), two pairs of primers were meticulously designed using Primer 5.0 software to amplify the entire *ZmNRT2.5* gene (Table S3), consisting of a 261‐bp 5′ untranslated region (5′ UTR), the 1564‐bp coding sequence (*ZmNRT2.5* has a single exon and no intron), and a 176‐bp 3′ UTR. These sequences were assembled and aligned using MEGA 11.0 software (available at http://megasoftware.net/). The association between polymorphic sites (SNPs and InDels) within all aligned sequences was evaluated using TASSEL 5.0 software, applying a standard MLM with a MAF of 0.05.

### RNA extraction, cDNA preparation and RT‐qPCR analysis

Total RNA was isolated from maize ears (about 5 cm) using an RNAprep Pure Plant Plus Kit (catalogue number DP441; Tiangen Biotech, China). Subsequently, 2 μg of total RNA was reverse‐transcribed into first‐strand cDNA using a PrimeScript II 1st Strand cDNA Synthesis Kit (catalogue number 6210A; Takara, Japan). Quantitative PCR (qPCR) was conducted using TB Green Premix Ex Taq (catalogue number RR420A; Takara) on a Chromo4 real‐time PCR detection system, following the manufacturer's guidelines (Bio‐Rad CFX96). Data analysis was performed using Opticon Monitor software (Bio‐Rad). For each gene, three technical replicates were conducted for each of three biological replicates. The maize *Ubiquitin1* gene was employed as the internal standard for normalisation. A comprehensive list of the primers used for the reverse transcription (RT)‐qPCR assays is provided in Table S3.

### Subcellular localisation of ZmNRT2.5

The full‐length coding sequence of *ZmNRT2.5* was cloned into the pGreen‐35S:eGFP vector to generate the *35S:ZmNRT2.5*‐*eGFP* construct. The primer pairs used for PCR amplification are listed in Table S3. Plasmid extraction and purification were carried out using a Plasmid Maxi Kit (Aidlab; PL1301) and a DNA Pure‐Spin Kit (Aidlab; DR0201), following the manufacturer's instructions. Maize protoplasts were isolated and transfected using previously established methods (Yoo *et al*., 2007).

To confirm the subcellular localisation of ZmNRT2.5, the full‐length coding sequence of *ZmNRT2.5* was cloned into the vector pSuper1300‐GFP to generate the construct *35S:GFP*‐*ZmNRT2.5*. The primers used are listed in Table S3. The construct was expressed in *Nicotiana benthamiana* leaves via Agrobacterium (*Agrobacterium tumefaciens*)‐mediated infiltration using strain GV3101, as described by An *et al*. (2017). Fluorescence imaging was performed using an LSM 900 confocal microscope (Zeiss, Germany).

### Promoter:GUS assays

A 2200‐bp promoter region upstream of the *ZmNRT2.5* translation start codon and a 281‐bp 3′ UTR fragment were amplified using KOD ‐Plus‐ DNA polymerase (TOYOBO, www.toyobo‐global.com) from B73 genomic DNA as the template. The PCR product was cloned into the pBEN‐MCS‐GUS vector, and its correct insertion was verified by Sanger sequencing. The specific primers used are listed in Table S3. The resulting *ZmNRT2.5pro:GUS* plasmid was transformed into the maize inbred line ND101. The seed materials were created by Center for Crop Functional Genomics and Molecular Breeding of China Agricultural University. For GUS staining, tissues including culms, tassel and ears of various ages were harvested from *ZmNRT2.5pro:GUS* transgenic maize and immersed in X‐gluc (5‐bromo‐4‐chloro‐3‐indolyl‐β‐d‐glucuronic acid/cyclohexyl ammonium salt) staining solution (GUS staining kit; GT0391; Huayueyang, Beijing, China). After incubation in the staining solution for 24 h at 37°C, the samples were dehydrated in 70% (v/v) ethanol for 3 h to remove chlorophylls. The GUS‐stained tissues were examined under a stereo microscope (Olympus SZX16) and photographed with a digital camera (Nikon D700).

### Transgenic maize construction and growth conditions

To generate *ZmNRT2.5* knockout lines by clustered regularly interspaced short palindromic repeats (CRISPR)/CRISPR‐associated nuclease 9 (Cas9)‐mediated gene editing, a specific 20‐bp single guide RNA (sgRNA) targeting the single exon of *ZmNRT2.5* was cloned into the pXUE411C‐BG vector backbone (Xing *et al*., 2014). The resulting construct was then introduced into maize inbred line ND101 through Agrobacterium‐mediated transformation, as detailed previously (Zhu *et al*., 2016). Briefly, immature embryos from the maize inbred line ND101 were transformed with the gene editing construct by the Center for Crop Functional Genomics and Molecular Breeding of China Agricultural University. Transgenic plants were identified by PCR and sequenced to detect mutations. The primers used for PCR genotyping and sequencing of the *Zmnrt2.5* mutant alleles are listed in Table S3. The ethyl methane sulfonate (EMS) mutant line (*Zmnrt2.5‐2*) was obtained from http://maizeems.qlnu.edu.cn/. The CRISPR line (*Zmnrt2.5‐1*) and EMS mutant line (*Zmnrt2.5‐2*) were backcrossed to their corresponding ND101 or B73 inbred lines three times. The phenotypes of *Zmnrt2.5‐1*, *Zmnrt2.5‐2* and their corresponding wild‐type sibling controls were investigated in field trials conducted at Beijing (Shangzhuang Experiment Field of China Agricultural University, 39.9° N, 116.3° E) and Tieling (42.3° N, 123.8° E) in 2020. The field experiments followed a randomised block design with three replicates. Each block contained three rows. Each row was 5 m in length with a spacing of 0.25 m between plants.

### Scanning electron microscopy (SEM) analysis

Pollen‐receptive ears, approximately 20 days after pollination (DAP), were harvested, and the outermost third layer of the husk leaf, progressing from the exterior to the interior, was partially excised. The abaxial epidermal surfaces of the central region of the husk leaf were examined using a scanning electron microscope (Hitachi TM3000; Hitachi High Technologies Corporation, Tokyo, Japan). Ten distinct areas within the central section of each husk leaf were selected for SEM examination.

### Determination of cell width and cell number

To characterise the husk leaves at the corresponding stages of SEM analysis, the entire digital image of the third husk leaf was employed to determine the width of constituent cells using IMAGEJ software. Cell width in the middle region was measured from the SEM images. For each plant, about 10 SEM images were evaluated to calculate the cell width. The number of epidermal cells was determined by dividing the width of the imaged husk leaf by the corresponding mean cell width.

### Phylogenetic tree analysis

The amino acid sequences for ZmNRT2.5 and related proteins used for phylogenetic tree reconstruction were obtained from Ensembl Plants (http://plants.ensembl.org/index.html). MEGA 11.0 was used for sequence alignment and reconstruction of a neighbour‐joining tree, setting the number of bootstrap replicates to 1000 (Tamura *et al*., 2011).

### Luciferase complementation image assay

The full‐length sequences of *ZmNRT2.5* (Hap1 and Hap2) and *ZmNPF5* were cloned individually into the JW772 (CLUC) and JW771 (NLUC) (Zhang *et al*., 2015) vectors using a One Step Cloning mix (Yesen Biotech), respectively. The resulting constructs were then transformed into Agrobacterium strain GV3101. Subsequently, positive Agrobacterium colonies harbouring each plasmid were cultured in LB medium to an OD600 of 0.6–0.8, pelleted by centrifugation and resuspended in a buffer containing 10 mM methylester sulfonate, 10 mM MgCl_2_ and 150 mM acetosyringone at pH 5.7. The suspended cells were then infiltrated into the leaves of 5‐week‐old *Nicotiana benthamiana* plants in various combinations using a needleless syringe. After a 48‐h incubation period in a growth chamber set to a 16‐h light/8‐h dark photoperiod, the leaves were infiltrated with 1 mM d‐luciferin (Promega Corporation). The luciferase signals were captured using a Tanon‐5200 imaging system. These experiments were independently performed at least three times.

### Protein expression and GST pull‐down assays

To generate the *ZmNRT2.5‐GST* and *ZmNPF5‐MYC* expression vectors, the coding sequences of *ZmNRT2.5* (Hap1 and Hap2) or *ZmNPF5* were PCR‐amplified using the primer pairs ZmNRT2.5‐GST‐F/ZmNRT2.5‐GST‐R or ZmNPF5‐MYC‐F/ZmNPF5‐MYC‐R (Table S3), and then individually inserted into the Super1300‐GST and Super1300‐MYC vectors. Recombinant proteins were produced in the leaves of 5‐week‐old *Nicotiana benthamiana* plants. After a 48 h incubation period in a growth chamber set to a 16 h light/8 h dark photoperiod, tobacco leaves expressing GST‐tagged and MYC‐tagged proteins were homogenised in lysis buffer (25 mM Tris–HCl [pH 8.0], 150 mM NaCl, 1 mM EDTA, 10% glycerol, 1% Triton‐X‐100, 0.1% NP‐40, 1 mM Pefabloc cocktail).

For GST pull‐down assays, equal amounts of purified proteins (e.g. ZmNRT2.5‐Hap1‐GST and ZmNPF5‐MYC, ZmNRT2.5‐Hap2‐GST and ZmNPF5‐MYC, GST and ZmNPF5‐MYC) were mixed and incubated in GST binding columns for 4 h at 4°C. After 10 min of centrifugation at 8000 ***g***, the mixed proteins were washed five times with washing buffer, eluted and detected through Western blot assays using an anti‐MYC or anti‐GST monoclonal antibody (ABclonal Biotechnology Co., Ltd., China).

### Bimolecular fluorescence complementation (BiFC) assay

The full‐length sequences of *ZmNRT2.5* (Hap1 and Hap2) and *ZmNPF5* were individually cloned into the pSPYNE vector (encoding the N‐terminal half of enhanced yellow fluorescent protein [eYFP; amino acids 1–155]) or the pSPYCE vector (encoding the C‐terminal half of eYFP [amino acids 156–239]) (Waadt *et al*., 2008). The resulting constructs were then transfected into maize protoplasts as appropriate pairs, and the transfected protoplasts were incubated at room temperature for 14–16 h. Fluorescence signals were observed using a laser confocal microscope (LSM 880; Zeiss, Germany).

### Field cultivation under high‐nitrogen and low‐nitrogen conditions

Plants from the *Zmnrt2.5‐1* mutant and wild type in the ND101 background were cultivated in the field in 2021 at the Zhuozhou Teaching Experimental Station of China Agricultural University. A mixture of nitrate (provided as KNO_3_) and ammonium (provided as (NH_4_)_2_SO_4_) (60% nitrate combined with 40% ammonium, w/w) was used as the nitrogen source, with an application rate of 1 kg N/100 m^2^ for the low‐nitrogen condition and 2 kg N/100 m^2^ for the high‐nitrogen condition for the entire growth period. The plants were arranged in 36 rows, with 10 plants per plot, and three replicates were set up for each nitrogen condition.

### Measurement of NO_3_ ^−^ content

To assess the grain yield and NO_3_^−^ content of the *Zmnrt2.5‐1* gene‐edited mutant in the ZD958 background, field trials were carried out during 2022 and 2023 at the Shangzhuang Experimental Station of China Agricultural University in Beijing. The planting density was 12 rows with 117 plants, with a spacing between maize plants of 20 cm (row space) and 15 cm (plant space). At maturity, ears with perfect kernel setting were harvested in each block and air‐dried to measure grain yield, hundred‐kernel weight, kernel number per row and kernel row number.

Wild‐type and *Zmnrt2.5‐1* mutant kernels and husk leaves from homozygous ears at 20 DAP were collected. For each sample, 0.2 g of fresh weight was used to measure the NO_3_^−^ content using a Plant Nitrate Nitrogen Test Kit (Suzhou Comin Biotechnology Co., Ltd. 100T/96S, Suzhou, China). All measurements were conducted on three biological replicates. The standard curve is described by the equation:

$$y=0.007034x+0.01704,R^{2}=0.99575$$

The formula to calculate the nitrate (NO_3_^−^) content was:

$$N{O_{3}}^{-}\;\text{content}\;\left(\mathrm{mg}/\mathrm{kg}\right)=\frac{\left(\Delta A\;-\;0.01704\right)}{0.007034}\times \left(\frac{V}{W}\right)$$

where Δ *A* is the absorbance value, *W* is the weight of the sample (g) and *V* is the volume of the extraction solution added to the sample (1 mL).

### RNA‐seq analysis

Total RNA was extracted from the ear tissue of the *Zmnrt2.5‐1* gene‐edited mutant and its wild type at the V12 stage utilising an RNAprep Pure Plant Plus Kit (DP441; Tiangen Biotech, China), following the manufacturer's guidelines. Both genotypes were cultivated in a greenhouse under high‐nitrogen (HN) and low‐nitrogen (LN) conditions. For each genotype, nine distinct ears were harvested, resulting in three independent biological replicates. The above RNA samples were subjected to Illumina‐based sequencing on a NovaSeq 6000 platform (Illumina). The raw sequencing reads were processed with TRIMMOMATIC (Bolger *et al*., 2014) to eliminate adapter sequences and low‐quality reads (*Q* score < 20). Subsequently, the clean reads were aligned to the maize reference genome (B73 RefGen_v4, AGPv4) using HISAT2 (v.2.2.0) with default parameters (Pertea *et al*., 2016). The read counts for each gene were obtained using FeatureCounts (v.2.0.1) (Liao *et al*., 2014). Differential expression analysis was conducted using the DESeq2 (v.1.30.0) R package (Love *et al*., 2014). Differentially expressed genes (DEGs) were identified using the criteria of *P* < 0.05 and absolute log2(fold‐change) > 0.585. The calculation of fragments per kilobase million (FPKM) for each gene was achieved through an in‐house R script (https://github.com/fudiyi/code‐for‐FPKM). Gene ontology (GO) term enrichment analysis was performed on the DEGs using agriGO v2.062 (http://systemsbiology.cau.edu.cn/agriGOv2/download.php).

## Conflict of interest

The authors declare no competing interests.

## Authors contributions

Y.H., J.H.L and W.M.T conceived and supervised the project. Q.W. conducted the experiments. Q.W., M.W., A.A.X., J.Y.W. and Z.W. analysed the data. T.X., D.T.J. and M. L. manage the material planting. Q.W., J.H.L. and Y.H. prepared the manuscript. All authors read and approved the final manuscript.

## Supporting information


**Figure S1** Phylogenetic tree of the NRT2 protein family in plants.
**Figure S2** Expression pattern of *ZmNRT2.5* and analysis of ZmNRT2.5 localisation.
**Figure S3** Sequence analysis of ZmNRT2.5.
**Figure S4** *In vitro* pull‐down assay showing the interaction between ZmNRT2.5 and ZmNPF5.
**Figure S5** Natural variation of *ZmNPF5* is correlated with maize husk leaf width.
**Figure S6** Performance of agronomic traits in the *Zmnrt2.5–1* mutant and its wild‐type siblings.
**Figure S7** Performance of agronomic traits in the *Zmnrt2.5–2* mutant and its wild‐type siblings.
**Figure S8** Width of the third husk leaf in wild type, *Zmnrt2.5–1, rhw1‐1* single and double mutants and overexpression maize lines.
**Figure S9** Decrease in the ratio of husk leaf width and NO_3_^−^ content for *Zmnrt2.5–1* relative to the wild type under high‐nitrogen (HN) and low‐nitrogen (LN) conditions.
**Figure S10** Volcano plot of differentially expressed genes (DEGs).
**Figure S11** Performance of agronomic traits in the ZD958*‐Zmnrt2.5–1* mutant and its wild type.

**Table S1** List of candidate genes identified by GWAS for husk width in maize.

**Table S2** Summary of sequence variations in the *ZmNRT2.5* coding sequence.

**Table S3** List of PCR primers used in this study.

**Table S4** NO_3_^−^ content in the selected Hap1 and Hap2 inbred lines.

**Table S5** Maize 3rd husk leaf width in wild‐type and the *Zmnrt2.5‐1* mutant.

**Table S6** Maize 3rd husk leaf width in wild‐type and the *Zmnrt2.5‐2* mutant.

**Table S7** Genes upregulated or downregulated in the *Zmnrt2.5‐1* mutant under the HN condition relative to the wild type.

**Table S8** Genes upregulated or downregulated in the *Zmnrt2.5‐1* mutant under the LN condition relative to the wild type.

**Table S9** Seed protein content in selected Hap1 and Hap2 inbred lines.

**Table S10** Seed protein content in the *Zmnrt2.5* mutant.

**Table S11** Seed protein content in the ZD958‐*Zmnrt2.5‐1* mutant.

## Data Availability

All sequencing data generated in this study are available at the National Center for Biotechnology Information (http://www.ncbi.nlm.nih.gov/sra) at the Sequence Read Archive under accession number PRJNA1140906.
