# Supplementary material for: Natural variation in ZmNRT2.5 modulates husk leaf width and promotes seed protein content in maize
Source: Plant Biotechnol J. 2025 Jan 5;23(4):1039–52. doi: 10.1111/pbi.14559 (PMC11933875; doi:10.1111/pbi.14559)
Supplement: Supplementary file 1 — Figure S1 Phylogenetic tree of the NRT2 protein family in plants. Figure S2 Expression pattern of ZmNRT2.5 and analysis of ZmNRT2.5 localisation. Figure S3 Sequence analysis of ZmNRT2.5. Figure S4 In vitro pull‐down assay showing the interaction between ZmNRT2.5 and ZmNPF5. Figure S5 Natural variation of ZmNPF5 is correlated with maize husk leaf width. Figure S6 Performance of agronomic traits in the Zmnrt2.5–1 mutant and its wild‐type siblings. Figure S7 Performance of agronomic traits in the Zmnrt2.5–2 mutant and its wild‐type siblings. Figure S8 Width of the third husk leaf in wild type, Zmnrt2.5–1, rhw1‐1 single and double mutants and overexpression maize lines. Figure S9 Decrease in the ratio of husk leaf width and NO3 − content for Zmnrt2.5–1 relative to the wild type under high‐nitrogen (HN) and low‐nitrogen (LN) conditions. Figure S10 Volcano plot of differentially expressed genes (DEGs). Figure S11 Performance of agronomic traits in the ZD958‐Zmnrt2.5–1 mutant and its wild type. [file PBI-23-1039-s008.pdf]

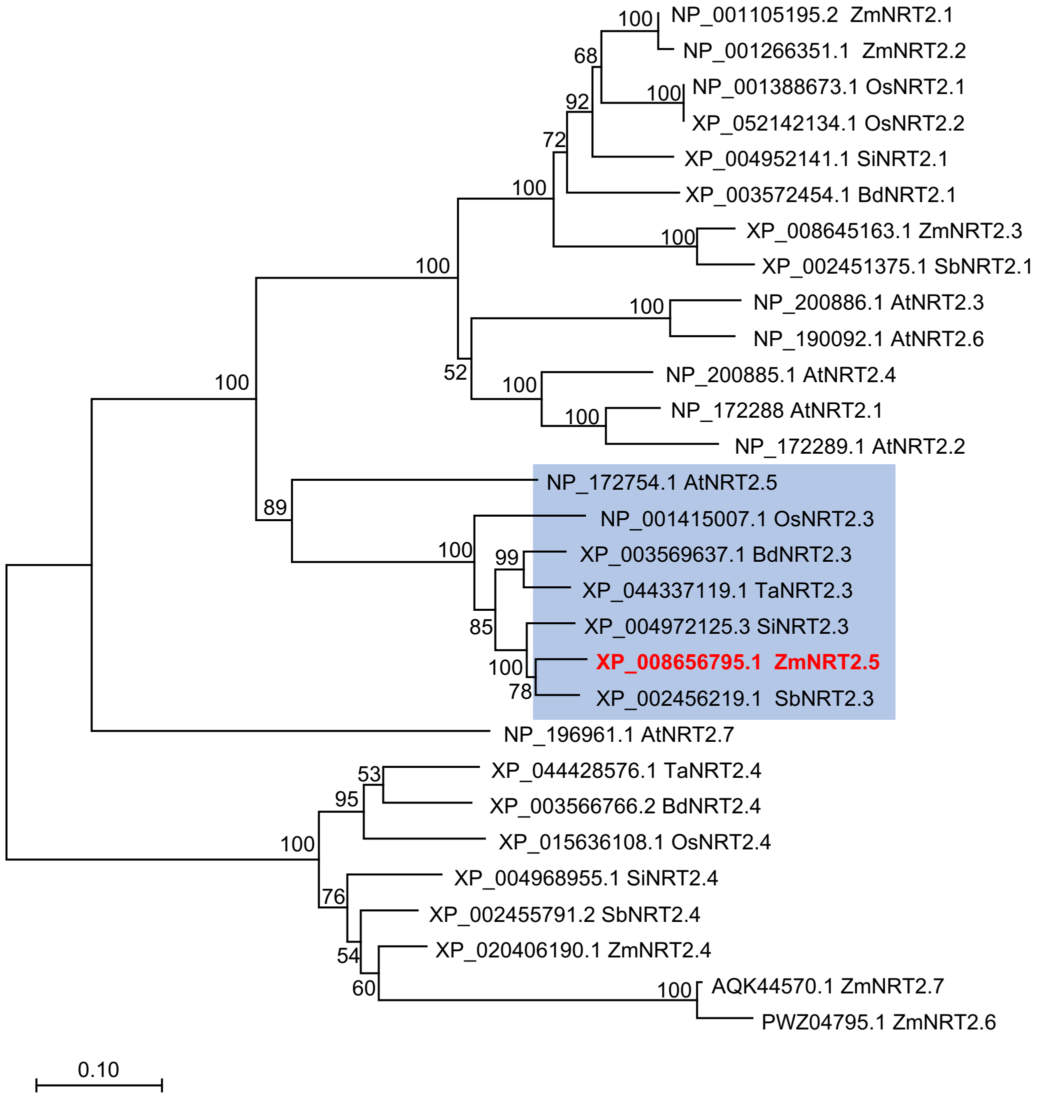

Supplemental Figure 1. Phylogenetic tree of the NRT2 protein family in plants.

A neighbor-joining tree was reconstructed based on the sequence alignment of full-length proteins in MEGA 11. ZmNRT2.5 is highlighted in red. The scale bar indicates the rate of substitution per site; the values at each branch point indicate the support from 1,000 bootstrap replicates.

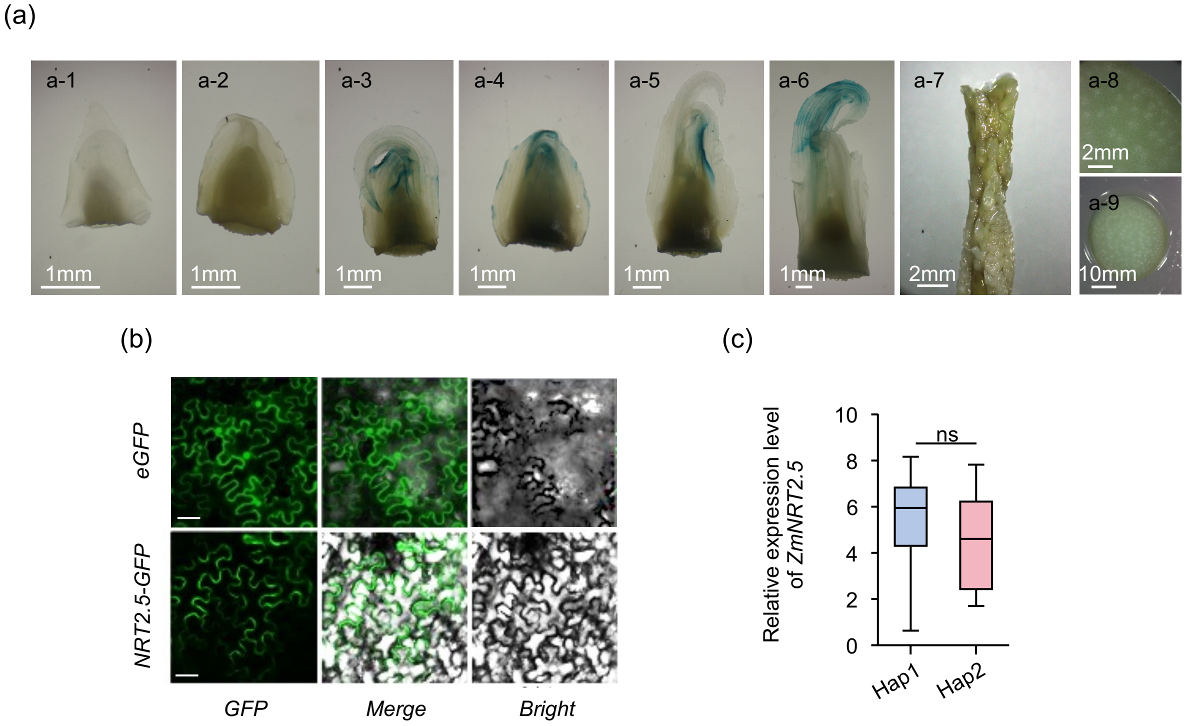

Supplemental Figure 2. Expression pattern of *ZmNRT2.5* and analysis of ZmNRT2.5 localization.

(a) (a-1–a-6) GUS staining of immature husks (2.0 mm [a-1], 2.5 mm [a-2], 5.0 mm [a-3], 6.0 mm [a-4], 8.0 mm [a-5], 17 mm [a-6]). Scale bars, 1 mm. (a-7) GUS staining of a tassel. Scale bar, 2 mm. (a-8, a-9) GUS staining of a stem. Scale bar, 2 mm (upper), 10 mm (lower). (b) Subcellular localization of free eGFP and ZmNRT2.5-eGFP in *N. benthamiana* epidermal cells via Agrobacterium-mediated infiltration of the indicated construct. Scale bars, 40 μm. (c) RT-qPCR analysis of *ZmNRT2.5* expression in inbred lines harboring the Hap1 or Hap2 haplotype of *ZmNRT2.5*. Data are presented as means ± SD from three independent biological replicates. Statistical significance was determined using the Wilcoxon rank-sum test. ns, not significant.

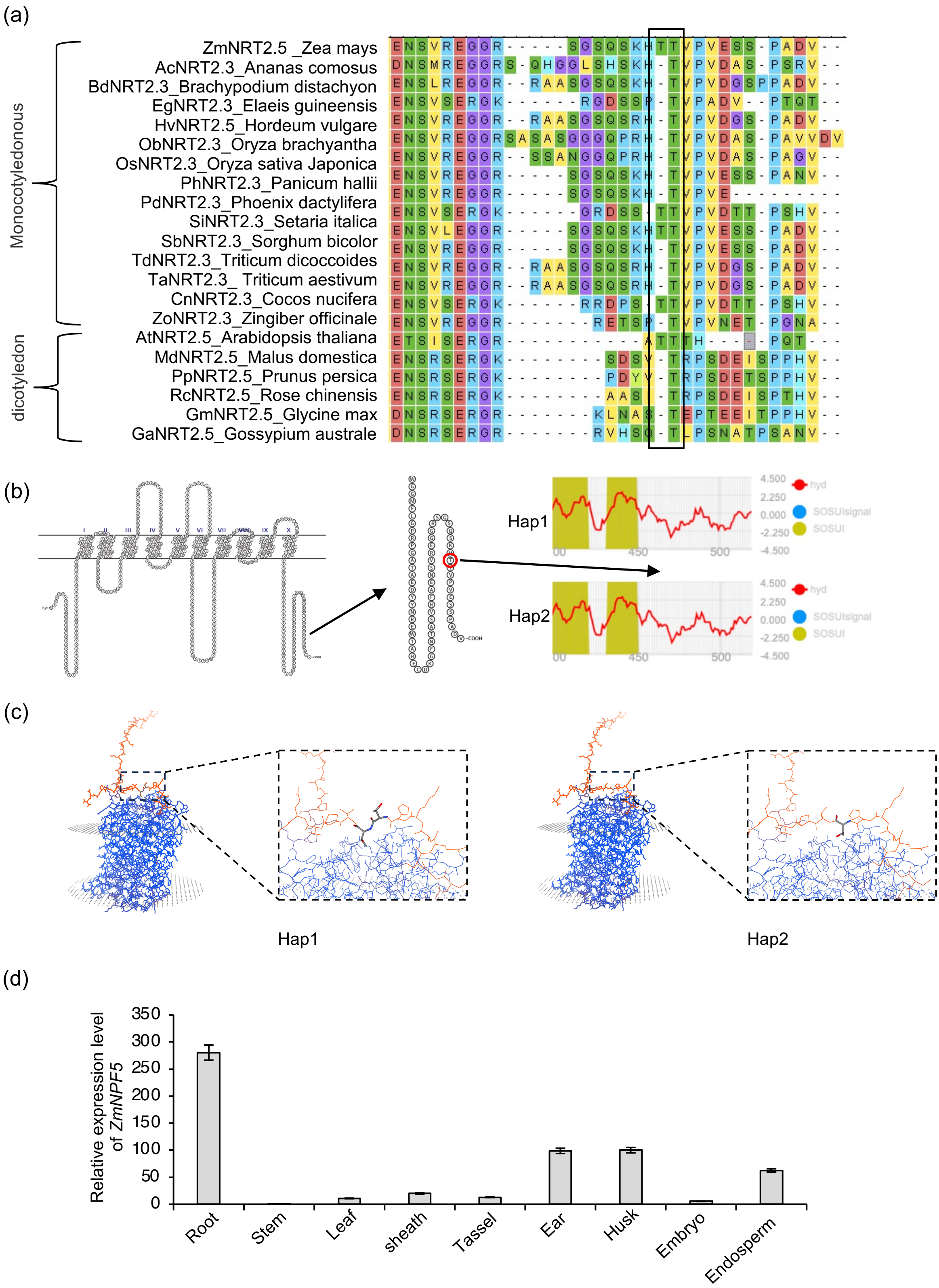
(a)
ZmNRT2.5 _Zea mays
AcNRT2.3_Ananas comosus
BdNRT2.3_Brachypodium distachyon
EgNRT2.3_Elaeis guineensis
HvNRT2.5_Hordeum vulgare
ObNRT2.3_Oryza brachyantha
OsNRT2.3_Oryza sativa Japonica
PhNRT2.3_Panicum hallii
PdNRT2.3_Phoenix dactylifera
SiNRT2.3_Setaria italica
SbNRT2.3_Sorghum bicolor
TdNRT2.3_Triticum dicoccoides
TaNRT2.3_ Triticum aestivum
CnNRT2.3_Cocos nucifera
ZoNRT2.3_Zingiber officinale
AtNRT2.5_Arabidopsis thaliana
MdNRT2.5_Malus domestica
PpNRT2.5_Prunus persica
RcNRT2.5_Rose chinensis
GmNRT2.5_Glycine max
GaNRT2.5_Gossypium australe
Monocotyledonous
dicotyledon
(b)
Hap1
Hap2
hyd
SOSUIsignal
SOSUI
4.500
2.250
0.000
-2.250
-4.500
450
500
(c)
Hap1
Hap2
(d)
Relative expression level of ZmNPF5
350
300
250
200
150
100
50
0
Root
Stem
Leaf
sheath
Tassel
Ear
Husk
Embryo
Endosperm

Supplemental Figure 3. Sequence analysis of ZmNRT2.5.

(a) The region affected by the InDel1604 domain multiple sequence alignment of ZmNRT2.5 and related proteins in monocotyledonous and dicotyledonous plants. NRT2.5 homologs were aligned using Muscle in MEGA 11.0 software. (b) Structure of ZmNRT2.5 (left) and prediction of hydrophilic structural differences between Hap1 and Hap2 (right) using the SOSUI program (engine ver.1.11; https://harrier.nagahama-i-bio.ac.jp/sosui/mobile/). (c) Prediction of the 3D structure of ZmNRT2.5 encoded by the Hap1 and Hap2 alleles by SWISS-MODEL (https://swissmodel.expasy.org/). The boxed area indicate the differing structures predicted by AlphaFold. (d) RT-qPCR analysis of *ZmNPF5* expression levels in various tissues. Data are presented as means ± SD; statistical significance was determined using the Student's *t*-test.

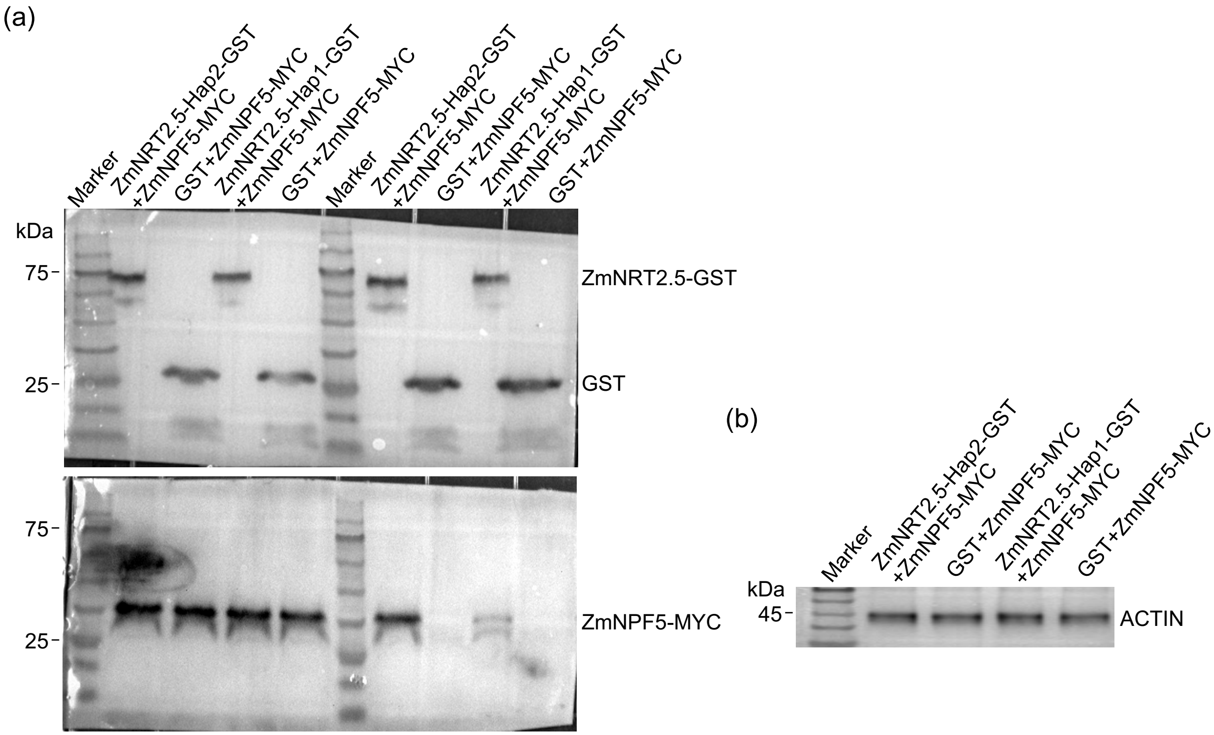

Supplemental Figure 4. ***In vitro*** **pull-down assay showing the interaction between ZmNRT2.5 and ZmNPF5.**

(a) Original uncropped and unadjusted western blots of Figure 2e. Western blot assays using an anti-GST antibody (upper) or an anti-MYC antibody (lower). (b) ACTIN was used as a loading control.

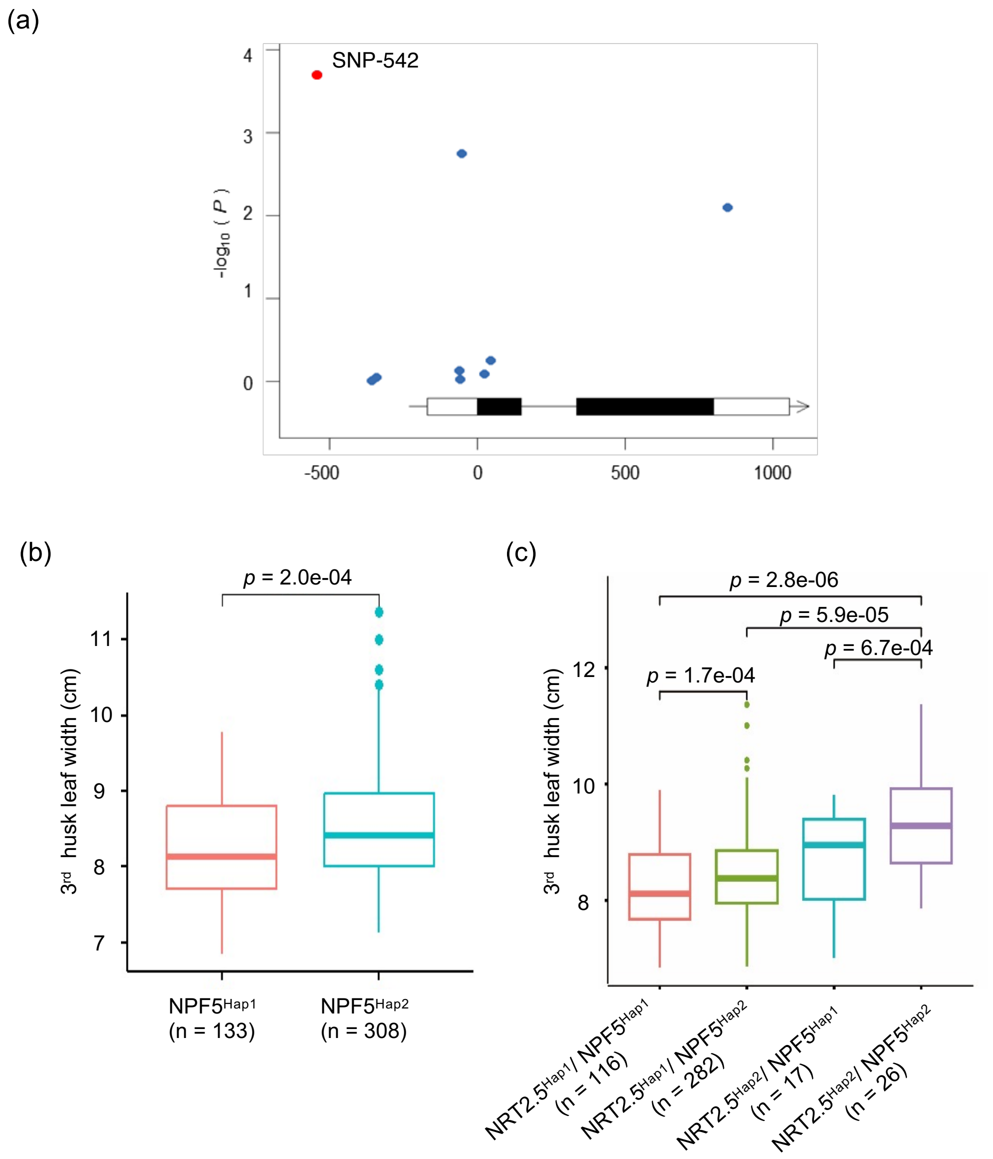

Supplemental Figure 5. Natural variation of *ZmNPF5* is correlated with maize husk leaf width.

(a) Regional Manhattan plot of the Zm00001d017095 (*ZmNPF5*) genomic region. The most statistically significant SNP is highlighted in red. (b) Width of the third husk leaf for each haplotype group of ZmNPF5. Statistical significance was determined using the Wilcoxon rank-sum test. (c) Width of the third husk leaf in the indicated samples. Statistical significance was determined using the Wilcoxon rank-sum test.

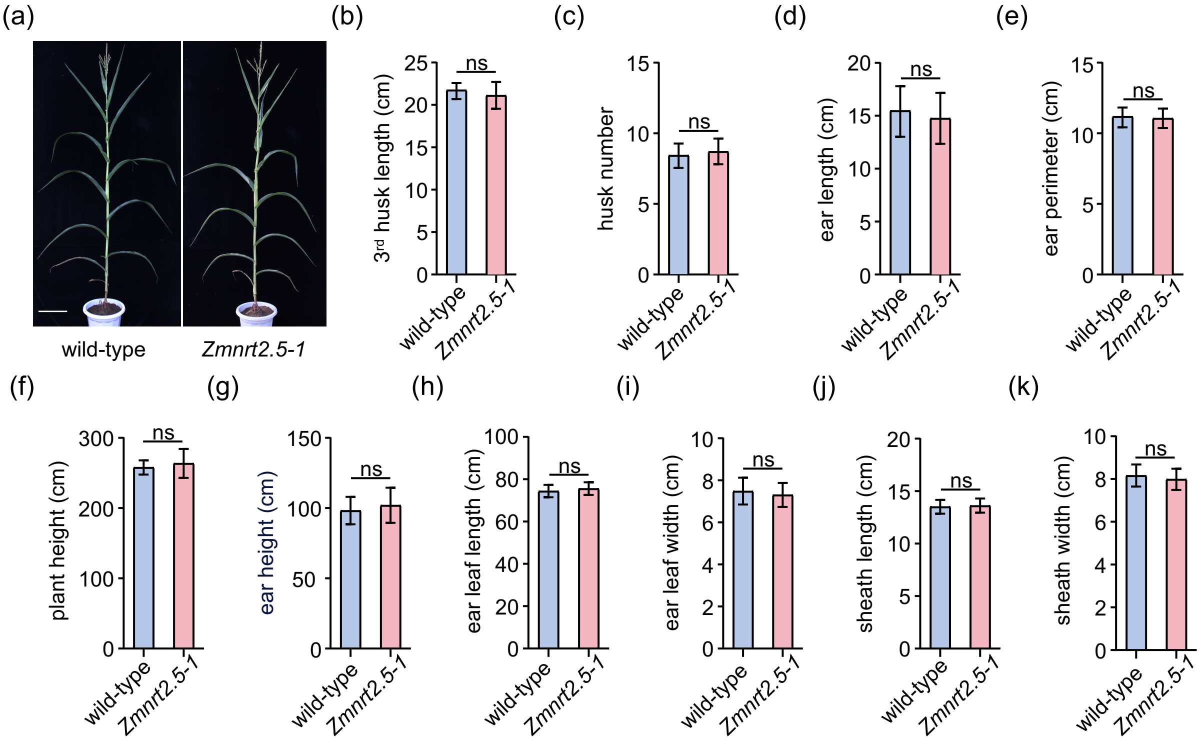

Supplemental Figure 6. Performance of agronomic traits in the *Zmnrt2.5-1* mutant and its wild-type siblings.

(a) Representative photograph of wild-type and *Zmnrt2.5-1* in the ND101 genetic background at 20 days after pollination (DAP). Scale bar, 20 cm. (b, c) Length of third husk leaf (b) and husk number (c) in wild-type and *Zmnrt2.5-1*. (d, e) Ear length (d) and ear perimeter (e) in wild-type and *Zmnrt2.5-1*. (f) Plant height of wild-type and *Zmnrt2.5-1*. (g–i) Ear height (g), ear leaf length (h), and ear leaf width (i) in wild-type and *Zmnrt2.5-1.* (j, k) Sheath length (j) and sheath width (k) in wild-type and *Zmnrt2.5-1*. Data are presented as means ± SD; statistical significance was determined using the Student's *t*-test. *n* = 57 and 55, respectively. ns, not significant.

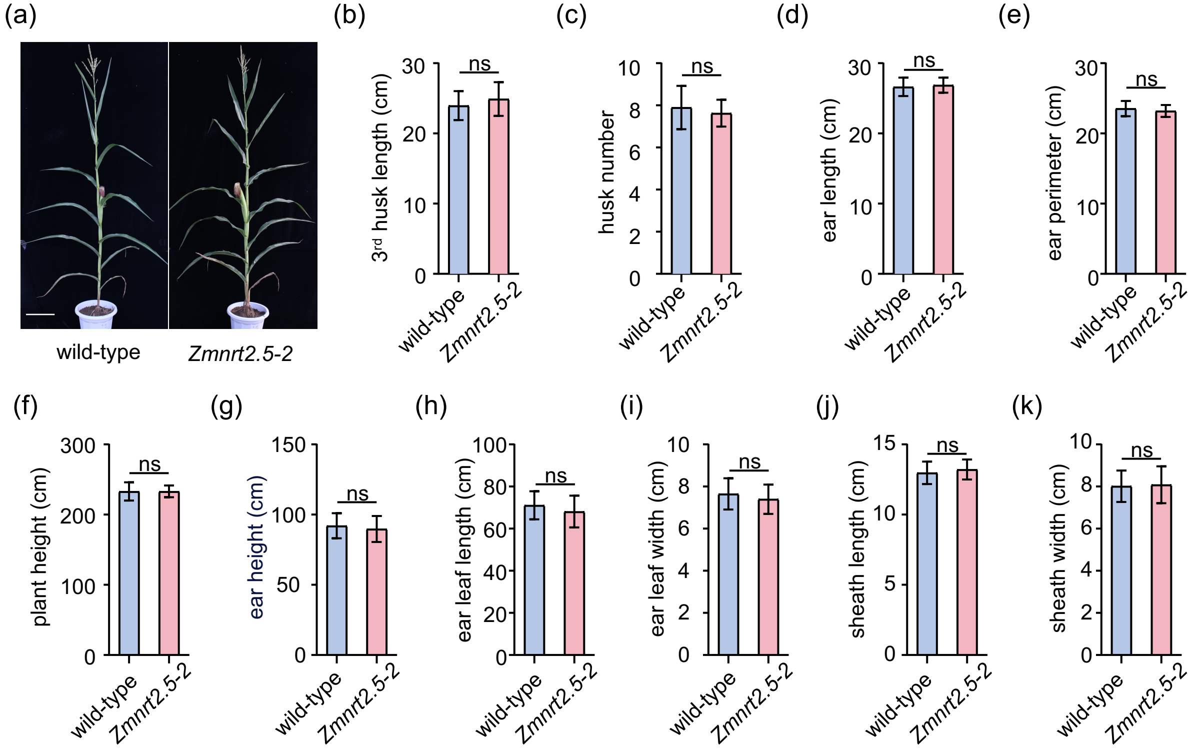

Supplemental Figure 7. Performance of agronomic traits in the *Zmnrt2.5-2* mutant and its wild-type siblings.

(a) Representative photograph of wild-type and *Zmnrt2.5-2* in the B73 genetic background at 20 days after pollination (DAP). Scale bar, 20 cm. (b, c) Length of third husk leaf (b) and husk number (c) in wild-type and *Zmnrt2.5-2*. (d, e) Ear length (d) and ear perimeter (e) in wild-type and *Zmnrt2.5-2*. (f) Plant height in wild-type and *Zmnrt2.5-2*. (g–i) Ear height (g), ear leaf length (h), and ear leaf width (i) in wild-type and *Zmnrt2.5-2*. (j, k) Sheath length (j) and sheath width (k) in wild-type and *Zmnrt2.5-2*. Data are presented as means ± SD; statistical significance was determined using the Student's *t*-test. *n* = 40 and 48, respectively. ns, not significant.

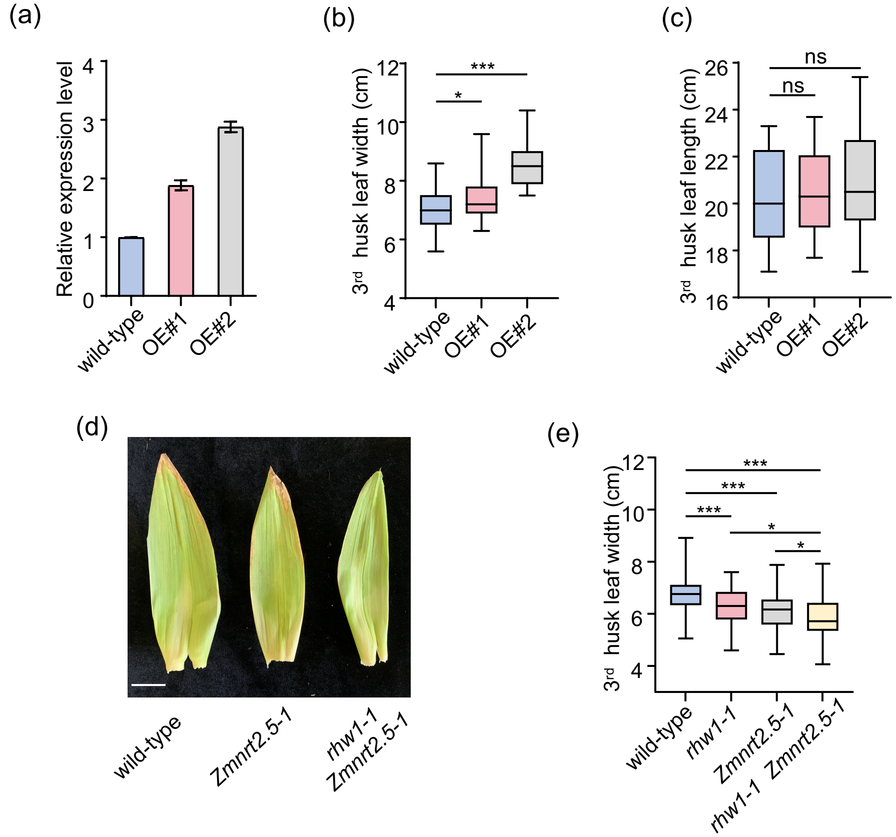

Supplemental Figure 8. Width of the third husk leaf in wild-type, *Zmnrt2.5-1, rhw1-1* single and double mutants, and overexpression maize lines.

(a) RT-qPCR validation of *ZmNRT2.5* expression in OE#1 and OE#2 relative to wild-type. Data are presented as means ± SD from three independent biological replicates. (b-c) Comparison of husk leaf width (b) and husk leaf length (c) between OE#1, OE#2 and wild-type plants. The width and length of husk leaves was measured at 20 DAP. n = 22 (wild-type), 23 (OE#1) and 21 (OE#2). Data are presented as means ± SD; statistical significance was determined using the Wilcoxon rank-sum test. $*P < 0.05$, $***P < 0.001$. ns, not significant. (d) The photograph of the third husk leaf of the *Zmnrt2.5-1*, *rhw1-1 Zmnrt2.5-1* double mutants and their corresponding wild-type (ND101) plants. Scale bar, 100 mm. (e) The width of third husk leaf of the *Zmnrt2.5-1*, *rhw1-1,* *rhw1-1 Zmnrt2.5-1* double mutants and their corresponding wild-type was measured at 20 DAP. n = 53 (wild-type), 74 (*rhw1-1*), 72 (*Zmnrt2.5-1*), 67 (*rhw1-1 Zmnrt2.5-1*). Data are presented as means ± SD; statistical significance was determined using the Wilcoxon rank-sum test. $*P < 0.05$, $***P < 0.001$.

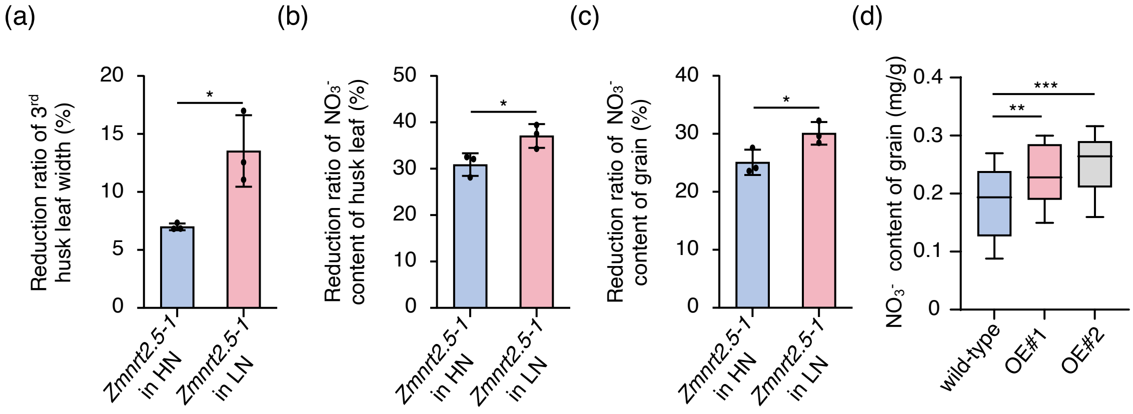

Supplemental Figure 9. Decrease in the ratio of husk leaf width and NO_3^- content for *Zmnrt2.5-1* relative to the wild-type under high-nitrogen (HN) and low-nitrogen (LN) conditions.

(a) Decrease in husk width in *Zmnrt2.5-1* relative to its wild-type when grown under high-nitrogen (HN) or low-nitrogen (LN) conditions (shown in Figure 4b). (b) Decrease in NO_3^- content of husk leaves from *Zmnrt2.5-1* relative to its wild-type when grown under HN or LN conditions (shown in Figure 4c). (c) Decrease in NO_3^- content of grains from *Zmnrt2.5-1* relative to its wild-type when grown under HN or LN conditions (shown in Figure 4d). Data are presented as means ± SD; statistical significance was determined using the Student's *t*-test. *$P < 0.05$. (d) NO_3^- content of grains between *ZmNRT2.5* OE#1, OE#2, and wild-type plants.

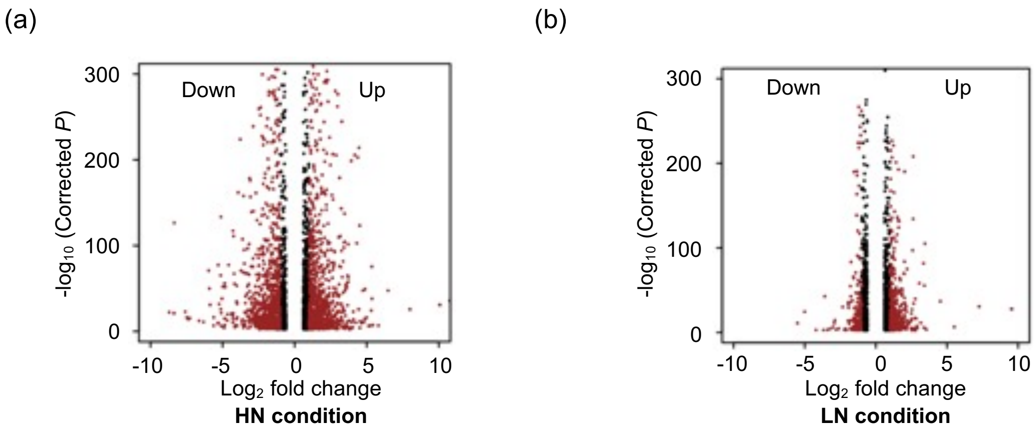

Supplemental Figure 10. Volcano plot of differentially expressed genes (DEGs).

(a) *Zmnrt2.5-1* compared to its wild-type under high-nitrogen (HN) conditions. (b) *Zmnrt2.5-1* compared to its wild-type under low-nitrogen (LN) conditions. The horizontal axis shows the $\log_2$ fold-change in gene expression in *Zmnrt2.5-1* relative to its wild-type; the vertical axis shows the statistical significance, as $-\log_{10}$(corrected *P*-value).

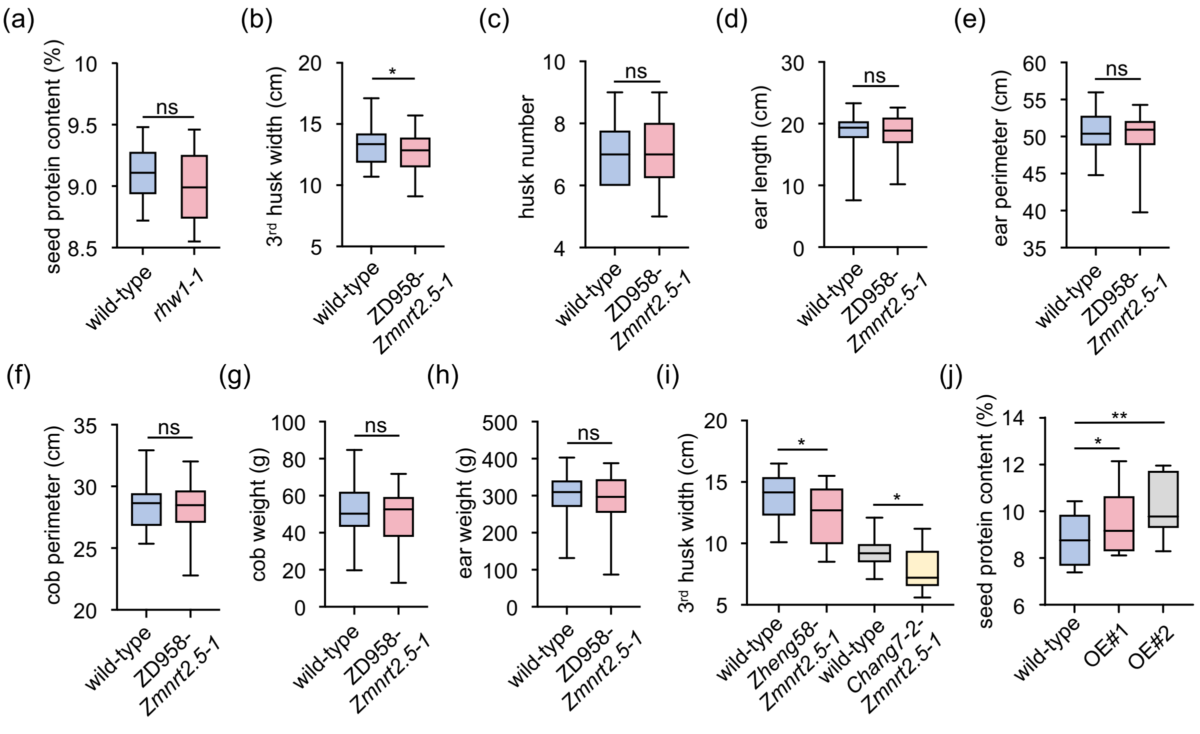

Supplemental Figure 11. Performance of agronomic traits in the ZD958-*Zmnrt2.5-1* mutant and its wild-type.

(a) Seed protein content of ZD958-*Zmnrt2.5-1* and its wild-type. (b, c) Width of the third husk width (b) and husk number (c) in ZD958-*Zmnrt2.5-1* mutant and its wild-type. (d, e) Ear length (d) and ear perimeter (e) of the ZD958-*Zmnrt2.5-1* mutant and its wild-type. (f, g) Cob perimeter (f) and cob weight (g) of the ZD958-*Zmnrt2.5-1* mutant and its wild-type. (h) Comparison of ear weight between ZD958-*nrt2.5-1* mutant and its wild-type. (i) Width of the third husk of the introgressed lines Zheng58-*Zmnrt2.5-1* and Chang7-2-*Zmnrt2.5-1* and their respective wild-types. Data are presented as means ± SD; statistical significance was determined using the Wilcoxon rank-sum test. ns, not significant, $*P < 0.05$. $n = 57$ and 55, respectively. (j) Seed protein content of *ZmNRT2.5* OE#1, OE#2, and their wild-type. Data are presented as means ± SD; statistical significance was determined using the Wilcoxon rank-sum test. $*P < 0.05$, $**P<0.01$. $n = 20$ respectively.
